# Goat productivity under smallholder farmer management in semi-arid and hot-humid parts of Zimbabwe: a useful step for building resilient goat production systems in marginalized communities

**DOI:** 10.1007/s11250-026-05215-2

**Published:** 2026-07-31

**Authors:** Anderson Munengwa, Emmanuel T. Nyahangare, Prosper Jambwa, Alban Mugoti, Stephen Mandara, Melody Dzviti, Nation Chikumba, Lyndy J. McGaw

**Affiliations:** 1 Phytomedicine Programme, Department of Paraclinical Sciences, Faculty of Veterinary Science, University of Pretoria, Private Bag X04, Onderstepoort, Pretoria 0110 South Africa; 2 Department of Livestock Sciences, Faculty of Agriculture Environment and Food Systems, University of Zimbabwe, P O Box MP167, Mt Pleasant, Harare, Zimbabwe; 3 Department of Veterinary Biosciences, Faculty of Veterinary Science, University of Zimbabwe, P O Box MP167, Mt Pleasant, Harare, Zimbabwe; 4 Department of Animal Production Sciences, Faculty of Plant and Animal Science and Technology, Marondera University of Agricultural Sciences and Technology, P O Box 35, Marondera, Zimbabwe

**Keywords:** Goats, Flock dynamics, Management practices, Production constraints, Smallholder farmers

## Abstract

Information about on-farm goat productivity patterns is crucial for building resilient production systems in marginalised communities. Goat flock dynamics, management practices and production constraints in selected smallholder farming systems of Zimbabwe were explored. A multistage sampling approach was adopted to recruit a total of 200 participants. The effect of environment on productive indices and constraints was analysed using the Mann-Whitney U test and either Ordinal or Nominal logistic regression, respectively, in IBM SPSS version 26. Results highlight the importance of goats to rural communities. On average, 10.01 ± 5.14 goats were maintained per capita and the largest proportion was found in hot-humid environments (*p* > 0.05). However, reproductive performance in terms of average gestation length (4. 47 ± 0.59 months), age at first service (11.20 ± 2.24 months) and kidding (15.67 ± 2.30 months) was poorer in semi-arid environments than in the hot-humid areas (*p* > 0.05). Moreover, optimum productivity is hampered by a multitude of factors, including selection of breeding animals mainly based on personal appraisal (Index = 0.120), dependence of animals primarily on natural forages for feed (Index = 0.213), sub-optimal housing conditions like irregular cleaning (Index = 0.123) and disease preponderance (Index = 0.129). Ailments were mainly controlled using ethnoveterinary medicines (Index = 0.103). Taken together, our results underline the crucial role of goats to rural livelihoods, which could be elevated even more through community-based capacity building on proper animal husbandry practices in terms of breeding, nutrition, health and housing management.

**Supplementary Information:**

The online version contains supplementary material available at https://doi.org/10.1007/s11250-026-05215-2.

## Introduction

Food is projected to be one of the major priorities for human survival in the 21st century due to drastic population increases (1.1% per year) and dwindling food resources (Ghosh et al. 2024). Nonetheless, the agricultural sector, including livestock production, is being threatened by the negative effects of climate change. Frequent dry spells and heat waves resulting from global warming are highly unfavourable to livestock as they interfere with metabolic processes (Reay et al. 2007). Moreso, these conditions increase water scarcity for animals to drink as well as fodder production and upscaling the preponderance of pathogens (Hegerl et al. 2007). As a result, poor farmers relying mainly on natural graze for their animals may perpetually fall victim to these consequences. Thus, any intervention strategy for production of resilient livestock species is relevant to the ongoing climate change situation. It is noteworthy that goats, unlike other livestock species, have resounding adaptive advantages that could be exploited to ensure a sustainable livestock production sector. This species of animal is capable of thriving under drought conditions, viz. intense hydric and heat stress (Azizi 2012). Accordingly, they can be reared even under the most disturbed environments which could otherwise be unsuitable for crop production. Similarly, by having high feed conversion efficiencies, limited greenhouse gas production levels, low start-up cost requirements and rapid turnover rates, these animals stand to be ideal vehicles for ameliorating the livelihoods of the poor (Zvinorova et al. 2016).

In southern Africa, goats are reared for various purposes, including meat (chevon), milk, generation of cash for meeting household needs and socio-cultural desires (Gwaze et al. 2009; Maburutse et al. 2012). Notwithstanding this, they continue to be sidelined in terms of research, as well as veterinary and extension support, often because their overall meat contribution at national level is thought to be insignificant (Mutibvu et al. 2015; Munengwa et al. 2020). However, this could realistically be due to a mere undervaluation of actual sales figures and slaughters as these are seldom recorded in rural areas harboring greater multitudes of the region’s goats (van Rooyen and Homann 2008). It is indeed opportune time that this marginalization of goats be revisited for the betterment of poor livelihoods. To drive this evolution, precise documentation of dynamics in flocks, management practices, constraints and production performances in these communal areas is a crucial preliminary step. This paves the way for future improvements in the sector so as to effectively fulfil its socio-cultural and/ or economic roles.

Even though research on strategies for upscaling the productivity of these important resources is evident, there have not been significant corresponding improvements (Ochan and Makosa 2020). It is worth noting that the relevance of any intervention strategy varies from farmer to farmer and time to time as largely influenced by factors of an ecological, social, economic and political nature (Ochan and Makosa 2020). Oftentimes, reluctance in taking cognizance of these factors in many small ruminant development initiatives is responsible for the associated letdowns (Kosgey et al. 2006). Additionally, management practices constantly change to keep abreast with dynamics in resource availability due to climate change. Consequently, for expansion of communal goat production systems, precise and detailed understanding of farmers’ day-to-day operational practices under different environmental conditions is necessary. These pieces of information help to guide stakeholders in making informed development decisions. Thus, the current study was designed to assess spatial variations in goat management, productivity and constraints under typical smallholder farming systems.

## Materials and methods

### Overview of study sites

**Fig. 1:**
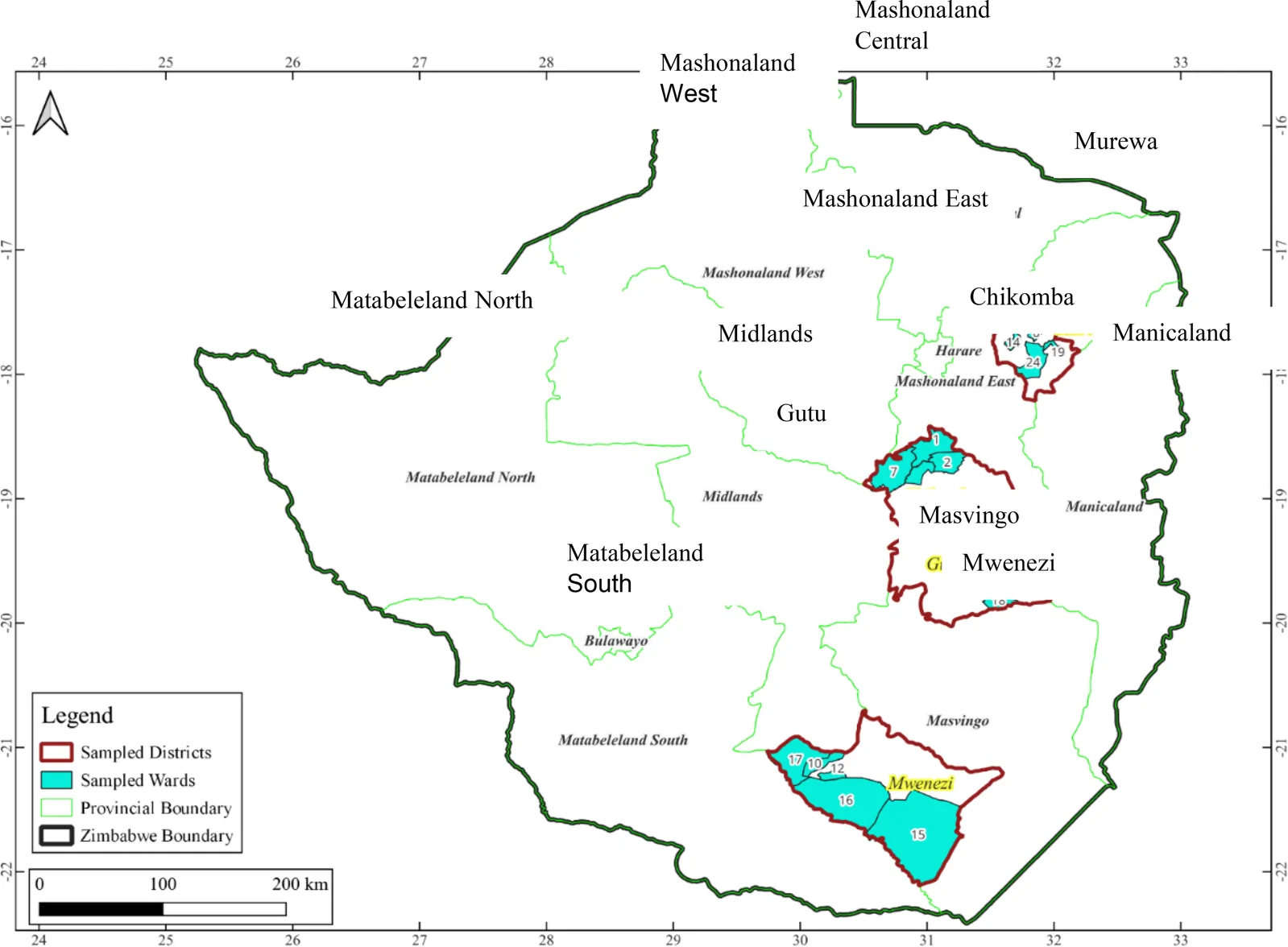
Map of the study area. Numbers in each district were village numbers for each sampled village

The research was conducted in four districts (Murewa, Chikomba, Gutu and Mwenezi) in Zimbabwe (Fig. 1). Murewa and Chikomba districts experience hot-humid climatic conditions with mean annual rainfall and temperature ranges of 700–800 mm and 18–28 °C, respectively (Chikodzi et al. 2013). These places are suitable for intensive crop and livestock farming as soils are naturally fertile and can support a wide diversity of vegetation. In contrast, Gutu and Mwenezi have typical semi-arid conditions (mean annual rainfall of ˂ 450 mm and temperature range of 14 to 29 °C) (Chikodzi et al. 2013). Soils in these two districts are generally poor and vegetation is mostly drought tolerant and sturdy. Extensive livestock rearing and game ranching is often done in these districts (Mugandani et al. 2012). In each district, 5 villages which maintained the highest number of goats according to the statistics from the district veterinary services department were purposively selected. Study sites are amongst the most resource-marginalized districts in the country.

### Study design, sampling, and ethical considerations

An explorative, qualitative research design was employed in the study according to Creswell (2003). To have a sample size that is representative of all the farmers in studied communities, a predetermined sample of 200 participants was initially calculated using the online Raosoft sample size calculator (Raosoft Inc 2004) using the following formula:

$$\mathrm{n}\,=\,\mathrm{Z}^2\times\mathrm{r}(100-\mathrm{r})/\mathrm{E}^2$$

Where,

n = Sample size

Z = Z- score corresponding to the desired confidence interval

r = Response distribution (50% expected variability in survey responses)

E = Margin of error (5%)

The representative value of 200 participants was guided by the selection of fifty participants from each of the four communities targeted. To ensure that only relevant participants who potentially have the required information were selected, a non-probability sampling procedure (purposive sampling) was employed. This was based on a set of criteria, viz. only farmers having attained a minimum age limit of 18 years, rearing of at least a unit of goats and willingness to participate in the study. The inclusion criteria for gathering goat productivity information by breed was as follows: for indigenous breeds, only the Mashona and Matebele were sampled while the Boer and the Kalahari red were the focus for exotic breeds. All goats falling within these breed groups from each participating farmer were automatically included in the study. These were chosen because they are the most preponderant breeds in studied communities (Mugoti et al. 2025) and it is thus plausible that farmers had detailed information pertaining to production and productivity of these breeds. Since the study was survey-based and animals were not directly involved, we strictly adhered to the principles of the declaration of Helsinki which stipulates mandatory informed consent, rigorous scientific protocols, risk minimization, and protection of vulnerable groups involved in the study. Ethics approval for this purpose was sought from the Faculty of Humanities, University of Pretoria (2296184-HUM004/1122). Field work was done between October 2022 and October 2023.

### Data collection

Face to face interviews were conducted in local languages of the communities (Shona and Ndebele) using validated semi-structured questionnaires (Online resource 1). Questionnaire validation was done according to multi-stage recommendations proposed by Creswell (2003). Firstly, face validity was ensured through a subjective assessment by survey experts and potential informants in such a way that the survey format, readability, and overall appearance was commensurate with the intended information. Secondly, content validation was done by field experts who rated the relevance and clarity of each question in the questionnaire so as to make sure that all aspects of the topic are covered on a content validity index. Thirdly, construct validity through a factor analysis determined the truthfulness of the survey in measuring the its intended theoretical construct. Fourthly, a pilot study covering 5% of the total study sample of goat keepers was conducted prior to the survey to identify weak, ambiguous, and/or irrelevant questions. Lastly, reliability testing was done to assess the survey’s consistency over time in terms of the questionnaire’s contents, the first part captured participants’ socio-demographic details such that only those meeting the selection criterion were included, viz. minimum age of 18 years and rearing of goats. The subsequent sections probed information on productivity patterns, production constraints and management practices.

### Data analysis

All statistical analysis was conducted in IBM SPSS, version 26 (IBM SPSS Statistics 2013). Since the data was not normally distributed, the non-parametric Mann-Whitney U test was used to unravel spatial variations in productivity variables between the two environments. Effects of production environment on constraints and management practices were analysed using either the ordinal or nominal logistic regression (depending on the data pattern) analyzed at 50% probability level. Ranking indices for ordered variables (production constraints and management practices) were calculated from the weighted mean scores of each variable according to Kosgey et al. (2006) as follows (step 1 to 2):

1 $$\text{Weighted mean}\,=\,\sum\,(\mathrm{n}_{1}\times2,\mathrm{n}_{2}\times1)$$

Where;

n = number of respondents with a given score (i.e. n_1_ = number of respondents with score1…n_2_ = number of respondents with score 2) and 1, 2, 3, 4 and 5 are the scores for each variable.

2 $$\begin{aligned}&\text{Ranking index score}\:\\&=\frac{\sum\:\begin{aligned}&[\left(3\times\:\mathrm{r}\mathrm{a}\mathrm{n}\mathrm{k}1\right)+\left(2\times\:\mathrm{r}\mathrm{a}\mathrm{n}\mathrm{k}2\right)\\&+\left(1\times\:\mathrm{r}\mathrm{a}\mathrm{n}\mathrm{k}3\right)]\mathrm{i}\mathrm{n}\mathrm{d}\mathrm{i}\mathrm{v}\mathrm{i}\mathrm{d}\mathrm{u}\mathrm{a}\mathrm{l}\:\mathrm{t}\mathrm{r}\mathrm{a}\mathrm{i}\mathrm{t}\end{aligned}}{\sum\:\begin{aligned}&[\left(3\times\:\mathrm{r}\mathrm{a}\mathrm{n}\mathrm{k}1\right)+\left(2\times\:\mathrm{r}\mathrm{a}\mathrm{n}\mathrm{k}2\right)\\&+\left(1\times\:\mathrm{r}\mathrm{a}\mathrm{n}\mathrm{k}3\right)]\mathrm{o}\mathrm{v}\mathrm{e}\mathrm{r}\mathrm{a}\mathrm{l}\mathrm{l}\:\mathrm{t}\mathrm{r}\mathrm{a}\mathrm{i}\mathrm{t}\mathrm{s}\end{aligned}}\end{aligned}$$

Here, it should be noted that all the responses on the 5-point Likert scale except for ‘Never’ were considered as yes and assigned a score of 2 and ‘Never’ was assigned a score of 1. This was applied on all ordered variables.

## Results

### Participant socio-demographic information

The majority of the households were male headed (62%). About 25% of the farmers had greater than 10 years of goat keeping experience. This was associated with flock sizes kept (*p* = 0.013). All participating farmers (100%) had at least some basic formal education.

### Goat flock sizes

Results revealed a substantial variability in goat flock sizes and structure between the two production environments (Table 1). At least every sampled farmer owned a unit of goats and flock sizes ranged from 1 to 33 goats with an average of 10.01 ± 5.14 goats per goat keeper. It is noteworthy that flock sizes differed by production region, viz. larger flocks were observed in semi-arid than in hot-humid environments (*p* = 0.002, Table 1). When compared by breed, there were more indigenous than exotic breeds (*p* = 0.001; Table 2).

**Table 1:** Effect of agro-ecology on selected flock compositional and production parameters

Variable	Mean	Std. Dev.	Min.	Max.	Mann-Whitney U test					
					Hot-humid		Semi-arid		U	Sig
					Mean rank	Sum of ranks	Mean rank	Sum of ranks		
Flock size (number)	10.01	5.14	1	33	83	8319	117	11781	3269	0.002^*^
No. breeding males	0.57	0.50	0	1	97	9700	104	10400	4650	0.321
No. breeding females	5.16	2.60	1	12	88	8859	112	11240	3809	0.003^*^
No. kids born per year	9.23	4.32	1	22	92	9200	109	10900	4150	0.042^*^
Litter size (number)	1.23	0.42	1	2	97	9750	103	10350	4700	0.321
No. parities per year	1.66	0.48	1	2	110	11,000	91	9100	4050	0.011^*^
No. females serviced	4.96	2.50	1	11	88	8806	112	11294	3756	0.002^*^
Age at first kidding (months)	15.67	2.30	12	20	63	6385	137	13714	1335	0.004^*^
Gestation length (months)	4.47	0.59	4	6	91	9122	109	10978	4072	0.011^*^
Age at first service (months)	11.20	2.24	8	16	66	6639	134	13460	1589	0.003^*^
No. kids surviving to weaning per year	8.16	3.73	0	18	91	9182	109	10918	4132	0.031^*^

^*^ Significant, *p* < 0.05

**Table 2:** Effect of goat breed on selected flock compositional and productivity parameters

Parameter	Mean	Std. Dev.	Min.	Max.	Mann-Whitney U test					
					Indigenous		Exotic		U	Sig
					Mean rank	Sum of ranks	Mean rank	Sum of ranks		
Flock size (number)	13.86	7.14	0	31	95	15344	125	4756	2141	0.001
No. kids born per year	9.23	4.32	1	22	91	14787	140	5314	1584	0.004
No. parities per year	1.66	0.48	1	2	95	15370	124	4730	2167	0.012
Age at first service (months)	11.20	2.24	8	16	97	15893	133	5893	2177	0.023
Age at first kidding (months)	15.67	2.30	12	20	98	16058	129	6903	2348	0.044
No. kids surviving to weaning	8.16	3.73	0	18	97	15661	117	4440	2458	0.051

NB: only parameters significantly influenced by breed at *p* < 0.05 are presented

### Selected goat productivity parameters

Comparative productivity data between agro-ecological production regions is presented in Table 2. The mean age at first kidding and service was 15 ± 2.30 and 11.20 ± 2.24 months, respectively. Servicing of female goats habitually occurred between February and March. The average gestation length was 4. 47 ± 0.59 months and litter sizes averaged to 1.23 ± 0.42 kids. Another noteworthy observation was the absence of breeding males in some households (min = 0; max 1) as opposed to at least a unit of does (min = 1; max 12). Nonetheless, the smallest proportion of farmers with breeding males maintained mating ratios of 1 buck:5 does. Goats in the hot-humid regions exhibited a shorter gestation length and attained age at first kidding and service earlier than those in the semi-arid regions (*p* < 0.05). This was also true for the number of kids surviving to weaning. In contrast, the average number of females serviced and kids born were higher in hot-humid communities than the semi-arid environments, albeit to breed differences (*p* < 0.05). The effect of breed type on productive performance was also documented (Table 2). Parameters such as average number of kids surviving to weaning, age at first service and kidding were higher for indigenous than exotic breeds (*p* < 0.05).

### Goat flock entries and exits

Table 3 presents goat flock entries and exits per farmer for the entire year in studied communities. Kids born on-farm were the major mode of flock entry (9.23 ± 4.32 goats/farmer/year) and varied with production environment (*p* = 0.014). Purchases, gifts-in and exchanges were seldom practiced (≤ 1 goats). Slaughters constituted the main mode of goat flock exit (mean = 1.71 ± 1.02 goats/ farmer/year). Deaths ensuing from parasites and diseases also immensely contributed to total exits (1.58 ± 1.35 goats/farmer/year). Incidences were more confined to hot-humid environments than the semi-arid regions (*p* = 0.001). Predators were also partly responsible for animal exits in semi-arid places (1.06 ± 0.18 goats/farmer/year).

**Table 3:** Flock entries and exits by agro-ecological region

Parameter (number)/year	Mean	Std. Dev.	Min.	Max.	Mann-Whitney U test					
					Hot-humid		Semi-arid		U	Sig
					Mean rank	Sum of ranks	Mean rank	Sum of ranks		
Total entries	12.86	1.92	1	27	90	9016	110	11084	3966	0.013^*^
Kidding	9.23	2.32	1	22	92	9200	109	10900	4150	0.014^*^
Purchases	1.13	0.94	0	5	102	10273	98	9826	4776	0.562
Gifts-in	0.97	0.74	0	2	88	8812	112	11288	3762	0.002^*^
Exchanges	0.93	0.07	0	3	90	9063	110	11037	4013	0.012^*^
Inheritance	0.62	0.01	0	5	101	10151	99	9949	4899	0.764
Total exits	6.02	1.30	1	13	106	10692	94	9407	4357	0.113
Slaughters	1.71	1.02	0	5	102	10292	98	9807	4757	0.532
Gifts out	0.52	0.72	0	2	97	9741	103	10359	4691	0.382
Deaths by diseases	1.58	0.35	0	5	131	13121	69	6978	1928	0.001^*^
Deaths by injuries	0.37	0.60	0	2	86	8610	114	11490	3560	0.064
Predation	1.06	0.18	0	4	90	9016	110	11083	3966	0.011^*^
Sales	0.78	0.02	0	4	94	9492	106	10607	4442	0.142

^*^ Significant, *p* < 0.05

### Goat production constraints

Table 4 presents a total of nine ranked production constraints reported by smallholder farmers to impact on optimum goat productivity. Mortalities due to parasites and diseases posed the biggest threat to goat production and productivity (Index = 0.129) followed by limited access to veterinary services (Index = 0.128) and feed shortages (Index = 0.127). Out of the nine documented constraints (Table 4), only five varied by production environment (*p* < 0.05) viz. access to: veterinary services, clean water, extension support, sufficient feeds and predators such as hyenas and pythons (Table 5). The odds of experiencing all these factors were higher in the semi-arid environments than the hot-humid areas (*p* < 0.014, Table 5).

**Table 4:** Goat production constraints faced by farmers (highest to least rank)

Variable	No. respondents		Weighted mean	Ranking score
	Yes	No		
Mortalities due to parasites and diseases	195	5	395	0.129
Poor access to veterinary services	192	8	392	0.128
Feed shortages	187	13	387	0.127
Predators	154	46	354	0.116
Lack of breeding males	126	74	326	0.107
Lack of production knowledge	110	90	310	0.102
Market constrains	101	99	301	0.099
Theft	96	104	296	0.097
Access to clean water	80	120	280	0.092

**Table 5:** Effect of agro-ecological region on constraints and management practices

Parameter	β(SE)	Wald’s χ2	Sig	Exp(β)	95% CI for Exp(β)	
					Lower	Upper
*Production constraints*						
Limited access to water	1.53(0.30)	26.47	0.002	4.62	2.58	8.27
Poor access to veterinary services	0.71(0.29)	6.13	0.014	2.04	1.16	3.60
Lack of production expertise	1.31(0.29)	20.61	0.001	3.69	2.10	6.47
Predators	0.78(0.28)	7.31	0.014	2.60	1.24	3.77
Shortage of feed	1.31(0.34)	14.37	0.003	3.69	1.88	7.25
*Management practices*						
Use of conventional de-wormers	-1.06(0.33)	10.49	0.003	0.35	0.18	0.66
Therapeutic use of conventional drugs	-0.58(0.29)	3.86	0.051	0.56	0.32	0.10
Use of medicinal plants	-0.59(0.11)	9.70	0.022	0.80	0.75	1.60
*Ad libitum* access to water	-0.49(0.21)	7.03	0.001	0.42	0.67	0.82

NB. Only significant variables at *p* < 0.05 are presented

### Goat flock management practices

#### Health management

Ethnoveterinary medicines were the major strategy of managing flock health, particularly for treatment of ailments (Index = 0.103) and control of helminth infections (Index = 0.091) (Table 6). This pattern was more common in semi-arid environments than the hot-humid regions by a coefficient of 0.80 times (*p* = 0.022; Table 5). It is noteworthy that farmers also reported that they destroy toxic plant species (Index = 0.092; Table 6). This was however common across production environments (*p* > 0.05). Veterinary service support in terms of access to orthodox drugs and veterinary advice on animal health management appears to be limited to a small proportion of the farmers, especially those in the hot-humid environments (*p* < 0.05, Table 5). A paltry proportion of the respondents claimed that they vaccinate goats against diseases (6%).

**Table 6:** Goat flock health management practices employed by farmers (highest to least rank)

Variable	No. respondents		Weighted mean	Ranking score
	Yes	No		
Treatment of sick animals with ethnoveterinary medicines	164	36	364	0.103
Deworming with ethnoveterinary medicines	133	67	333	0.091
Destroy toxic plants	108	92	308	0.092
Burning/ burying infected carcasses	94	106	294	0.082
Quarantine new additions to the flock for at least 30 days	85	115	285	0.084
Treatment of sick animals with conventional drugs	72	128	272	0.082
Deworming with orthodox drugs	69	131	269	0.074
Maintenance of foot baths	68	132	268	0.073
Dipping	62	138	262	0.072
Consultation of veterinarians for medical advice	54	146	254	0.071
Keep a closed flock	40	160	240	0.070
Leaving diseased carcasses to decay in situ	20	180	220	0.063
Vaccination for diseases	12	188	212	0.062

#### Nutritional management

A total of six feeding strategies were reported in the study (Table 7). Natural grazing lands were reported to be the primary source of feed for goats across study sites (Index = 0.213). Supplementation with commercial feed supplements was seldom practiced. Notwithstanding the fact that goats had access to clean water (Index = 0.188), *ad libitum* access was seldom practiced in the semi-arid than hot-humid environments (*p* = 0.001; Table 5).

**Table 7:** Goat feeding management practices employed by farmers (highest to least rank)

Variable	No. respondents		Weighted mean	Ranking score
	Yes	No		
Natural grazing lands	143	57	343	0.213
Ensuring access to clean drinking water	103	97	303	0.188
*Ad libitum* provision of water	92	108	292	0.181
Conserved cut and carry feed	40	160	240	0.149
Feed supplementation during the dry season	18	182	218	0.135
Provision of commercial feed supplements	13	187	213	0.132

#### Housing management

**Fig. 2:**
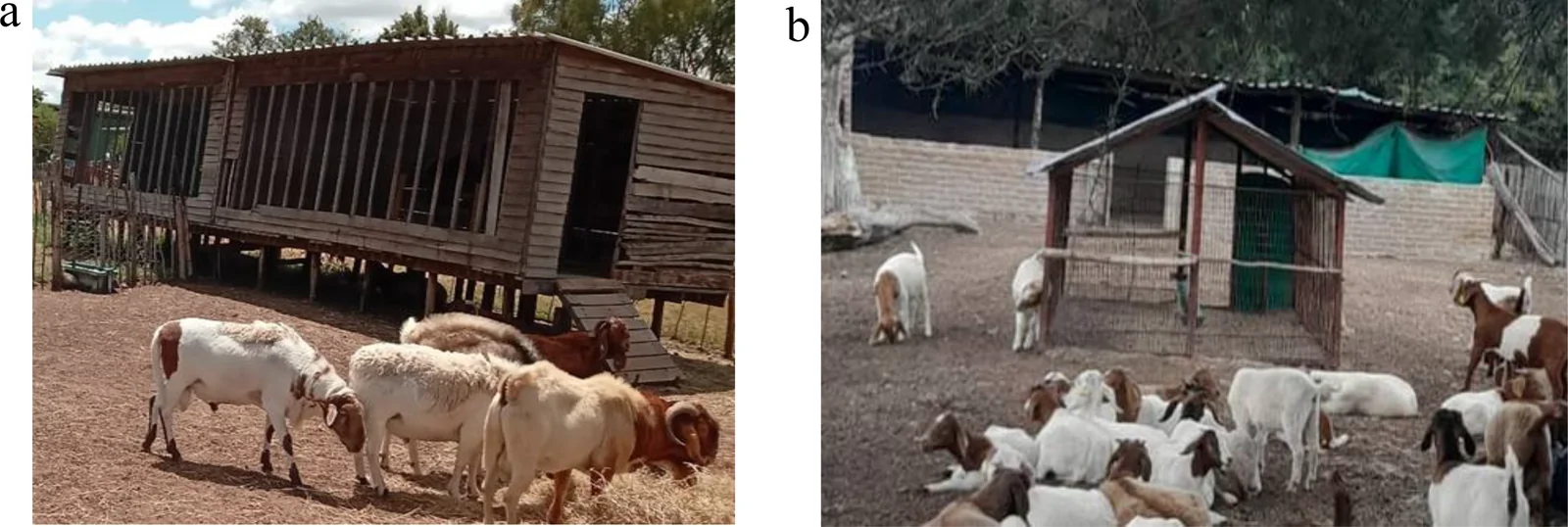
**a** Elevated goat pen. **b** Lean-to-type shed constructed directly on the ground surface

Two types of goat pens were reported in the study vis-à-vis (1) raised above the ground and (2) constructed directly on the ground surface (Table 8; Fig. 2a and b). Adequate ventilation was ensured in goat pens (Index = 0.222) while manure was not routinely removed from the pens (Index = 0.123). Despite the challenge of predators, particularly in the semi-arid environments, farmers rarely established predator barriers on or around goat pens (Index = 0.169). A small proportion (20%) of the goat keepers separated goat pens from those of other animals.

**Table 8:** Goat housing management practices employed by farmers (highest to least rank)

Variable	No. respondents		Weighted mean	Ranking score
	Yes	No		
Ensuring ventilation in pens	89	111	289	0.222
Housing pens raised above the ground	63	137	263	0.202
Separate houses from other animals	40	160	240	0.184
Predator barriers around pens	20	180	220	0.169
Removal of accumulated manure in pens at least monthly	10	190	290	0.123

#### Breeding management

A total of six breeding management strategies were documented in the study (Table 9). Of these, culling of unproductive goats (Index = 0.139) and castration of male goats less than 3 weeks of age (Index = 0.125) were often reported. For selection of breeding stock, farmers preferred the use of visual appraisal (Index = 0.120).

**Table 9:** Goat breeding strategies employed by farmers (Highest to least rank)

Variable	No. respondents		Weighted mean	Ranking score
	Yes	No		
Culling of unproductive goats	90	110	290	0.139
Castration of male animals of less than 3 weeks of age	60	140	260	0.125
Controlled mating	60	140	260	0.125
Selection of breeding stock based on visual appraisal	50	150	250	0.120
Seasonal restriction of mating	32	168	232	0.111
Selection of breeding stock using production records	28	172	228	0.110

## Discussion

In this particular study, socio-demographic data is of significance as it gives insights on specific groups of individuals who engage in routine management of the animals. This essentially increases the chances of obtaining useful information that could be used for improving productivity. Female representation among goat-keepers was low, based on gender proportions, which highlights the dominant role of males in undertaking routine livestock management activities and the potential of accumulating valuable knowledge about rearing of goats (Zvinorova et al. 2016; Mpofu et al. 2020). The observation that flock sizes maintained by farmers increased with increasing production experience is consistent with earlier reports (Maburutse et al. 2012; Zvinorova et al. 2016). It would logically follow that experienced farmers are important sources of practical goat keeping information as they supposedly provide better care of their animals. Additionally, the higher level of literacy reported in this study is likely to augment smooth information dissemination from extension services to farmers for the betterment of goat productivity.

The presence of at least a unit of goats in every sampled household is typical of African rural societies (Gwaze et al. 2009; Sikosana and Senda 2010). The high productivity of goat rearing in communal systems stems from their adaptability, socio-economic importance, market demand, and potential for increased output (Gwaze et al. 2009). Indeed, this serves as a genuine testimony to the fact that goat production is an important pillar of the livelihoods of poor communities. Nonetheless, when our data were compared with previous national flock holdings, it appears that there is a time-dependent progressive decline in goat populations from average flock sizes of 15 (Barret 1991), 12 (Kusina and Kusina 2002) and 10 goats (Mhlanga et al. 2018) per capita. It is therefore undoubtedly an opportune time for appropriate practical approaches to be employed for the enhancement of goat productivity. Larger goat flocks were observed in semi-arid areas compared to hot-humid communities, likely because goats are more resilient to harsh environments than other livestock (Gwaze et al. 2009; Zvinorova et al. 2016). Given the limited opportunities for crop farming in these arid regions, farmers rely on hardy livestock. Therefore, enhancing the productivity of indigenous goats which thrive under intense hydric stress and feed shortages is crucial for improving local livelihoods (Zvinorova et al. 2016). Moreover, native breeds could serve as a crucial genetic resource bank for adaptive traits that can be upgraded by high performing exotic breeds through cross breeding.

Reproductive performance in terms of the average age at first kidding and first service, gestation length and number of females serviced was poor in semi-arid agro-ecological environments than the hot-humid areas. For a doe to reach puberty and become sexually active, several morphological (biochemical and physiological) changes have to occur until ovulation occurs. These processes are known to be largely affected by nutrition and breed of the doe (Ochan and Makosa 2020). Most reproductive characteristics can be modulated by adapted nutrition which targets the different reproductive stages of the animal. Angora goats, for example, typically ovulate once per cycle under most production conditions but may have two under good nutritional conditions (Fatet et al. 2011). In tropical regions, goats often experience limited access to feed, both in terms of quality (nutrient profile) and quantity. This feed limitation is largely responsible for delayed sexual development, reductions in litter size, fertility and prolificacy (Warui et al. 2007; Fatet et al. 2011). This also appears to be the situation with animals involved in our study as comparative analysis of the reproductive performance within different environments revealed that reproductive wastage was more pronounced in semi-arid environments. With the harsh climatic conditions in the semi-arid environments which reduce animal feed resources, it is plausible that nutritional deficiencies and/or underfeeding could be some important environmental factors leading to observed trends. This is also supported by the tendency of goat-keepers to depend mostly on natural grazing conditions for animal feed with limited chances of supplementation with commercial and/or conserved hays.

When compared with similar local studies (Kusina and Kusina 2002; Mhlanga et al. 2018) our observed average age at first service and kidding was slightly delayed. Given the same environmental conditions in which these studies were conducted, it is possible that delays could have occurred due to factors other than or in conjunction with nutrition. One such possibility is the limited exposure of females to males as bucks were scarce in the current study. Continuous female exposure to males is known to stimulate reproduction through male pheromones which initiate a cascade of reproductive physiological reactions in does – a phenomenon regarded as the “buck effect” (Chemineau et al. 2006). The buck effect phenomenon can be elevated even more through nutritional supplementation. Fatet et al. (2011) documented that oestrous behaviour and rates of ovulation and pregnancy in response to the buck effect was increased in goats which received graded levels of feed supplements. With regards to litter size, the observed range of 1 to 2 is in harmony with the results reported elsewhere by Webb et al. (1999) and Elieser et al. (2012). Given that timed feed supplementation directly influences folliculogenesis - a critical step in developing fertilisable ova (Rashidi et al. 2011), optimizing ovulation rate this way presents a clear opportunity to increase litter size.

When our reproductive performance results were compared by breed it was apparent that exotic breeds outperformed indigenous breeds. Similar annotations with respect to the average age at first kidding and first service were reported in studies conducted in Botswana (Mpofu et al. 2002) and South Africa (Monkhei and Madibela 2005). To ensure robust applicability, future research should assess these reproductive benchmarks across broader subtropical environments, thereby establishing optimal, breed-specific conditions. Gestation length varied by dam breed, as also shown by Mellado et al. (2000), where Granadina goats had shorter pregnancies (149.0 ± 0.31 days) than Alpine goats (151.4 ± 0.46 days). The average gestation of 4.47 ± 0.59 months observed here aligns with findings from Webb et al. (1999) and Mhlanga et al. (2018), allowing for expected breed variations.

The tendency of servicing female goats in at least one of the three months of the breeding season i.e. February to March could be linked to the availability of feed resources during the rainy season such that most of the females were in a well-fed state and easily attained heat. However, it follows that most of the kids were born during the winter season when temperatures were low and life-threatening under sub-optimum shelter conditions. Moreso, it is highly likely that dams giving birth during this period experienced severe energy restrictions which could not meet the physiological requirements for late pregnancy and lactation. Previous research has documented that severe nutritional restriction during late pregnancy reduces kid birth weight and survival (Kenyon and Blair 2014) and under extreme feed deficiency cases, kid survival will only be possible with intensive intervention (Redmer et al. 2004). The observed low kid survival rate in hot-humid environments was expected since the prevailing conditions are highly favourable for disease and parasite preponderance. In general, kid survival rate can be improved through breeding strategies and manipulation of environmental factors such as shelter conditions and nutrition. Following previous reports by Rashidi et al. (2011) that birth weight was positively correlated with kid survival, the selection of breeding sires with high breeding values for birth weight should result in increased kid survival rates. The same study also observed that there was a direct interplay between environmental factors during gestation and post weaning mortality. Improvements in feed quality and quantity (especially during the last six weeks of pregnancy and during lactation) should also improve kid survival rates. In the studied communities, goats relied almost exclusively on natural grazing, with virtually no supplementation during dry seasons. The energy and protein requirements of goats are determined by a complex interplay of biotic and abiotic factors, including breed, health, and thermoregulation (Mugoti et al. 2025). While a standard maintenance and low-production diet requires approximately 9.4 MJ/day of energy and 54 g/day of metabolizable protein (Rashidi et al. 2011), the low-energy, high-fiber natural forage in this study likely fails to meet these requirements. Although Rashidi et al. (2011) studied European breeds, these figures provide a necessary, though preliminary, benchmark for assessing African breeds (Mugoti et al. 2025). Energy is prioritized for maintenance, with surplus energy allocated to production and reproduction. This hierarchy likely explains the higher rates of reproductive wastage observed in semi-arid environments. The current situation emphasizes the need for adapted and targeted supplementation of feeds through conserved cut and carry hays, commercial feeds and forage legumes. Given the issue of costs and availability surrounding commercial feeds, browse plant species such as *Acacia karroo (Vachellia karroo)*, which are predominant even in semi-arid environments, could be used throughout the year (including the extended dry periods) to argument the feed (Brown et al. 2016) In general, most of the browse species are endowed with high levels per kilogram of dry matter protein (14 to 26% CP) and energy (11 to 14 MJ of ME), high levels of organic matter digestibility (50–60%) and noteworthy mineral levels (Brown et al. 2016). They could also be used to supplement low quality tropical forages and crop residues. Notwithstanding these interesting attributes, intake by goats is often hampered by secondary metabolites which act as antinutritional factors. Through research, ensiling them with forages adapted to disturbed environments could be a potential avenue for improving goat productivity in semi-arid environments. This also presents an interesting opportunity for habitual harvesting of tree pods and leaves for feed supplementation during periods of intense feed scarcity, a practice that has often been lacking in these studied communities. Water, as another crucial nutritional component, was readily available to animals although some level of scarcity was noticeable in semi-arid environments. Goats, even though they are regarded as well adapted to hydric stress, require consistent access to clean water for proper digestion, thermoregulation, and overall health. When water is scarce, dehydration can result in health problems and reduced milk production in lactating does (Mugoti et al. 2025). Water harvesting, especially during the rainy season for use in the dry season, can help to reduce this challenge.

With respect to breeding, our study highlights that there were no proper breeding programs followed by farmers. The observation that some households had complete absence of breeding males as opposed to the presence of at least a unit of does is in harmony with earlier reports of Mhlanga et al. (2018). It is highly possible that the limited proportion of male goats in the study may have been due to the fact that by virtue of their body size, males were often selected for marketing, culling during traditional ceremonies and exchanges with other commodities while females were left to complete their reproductive cycles. Nonetheless, the smallest proportion of farmers who had breeding males appear to underutilize them (1:5; buck: doe) when compared to the generally recommended mating ratio of 1 buck to 25 does. As a step to cover shortages of breeding males, bucks of genetic merit may be used to systematically service does of the community following the appropriate mating ratios. Common practices such as culling of unproductive goats and castration of bucks at less than 3 weeks of age are highly encouraging as they form a supportive framework for the controlled mating programs reported in visited areas. These have also been suggested among other strategies like pedigree recording and progeny testing and genomic characterization as potential drivers for sustainable goat breeding (Monau et al. 2020). However, the reported selection of breeding stock using visual assessment with no records is subjective and may lead to unfruitful breeding outcomes such as higher inbreeding coefficients (Elieser et al. 2012). Like in most developing countries, effective goat breeding programs have been mostly limited by lack of pedigree and performance records for traits of economic importance (Rashidi et al. 2011). One strategy to ensure sustainable breeding in these communal areas is to ensure a thorough capacity building of farmers and establishing a communally owned open nucleus breeding system from which bucks of genetic merit are selected and disseminated to breeding flocks within the community. The strategy has been successfully implemented with notable success in Ethiopia, Uganda and Malawi through funded projects from the United States Agency for International Development and the United States Department of Agriculture (Monau et al. 2020). We also suggest harmonization of the on-farm phenotypic selection programs with genotypic selection programs conducted under well-controlled conditions such as at a research centre. This way, selected bucks of genetic merit could be distributed to the goat populations in smallholder farms. Particular attention may be focused on traits such as feed efficiency, kid body weight, daily gain and mortality and performance attributes of does. These are also the major components of the profitability in extensive goat production systems (Rashidi et al. 2011).

Housing is an important aspect of goat production since it has a substantial impact on productivity, welfare, and health of goats (Mugoti et al. 2025). In this study, pens were either constructed directly on the ground surface or elevated from the ground. While most of the farmers preferred the lean-to type of shed constructed directly on the ground surface, poor drainage and the proliferation of vector-borne diseases remain serious concerns (Mugoti et al. 2025). This also explains why hygienic conditions, with regards to removal of accumulated faeces and urine in pens, was seldom practiced in studied communities as the type of pens made it difficult to conduct the removal process. Apart from the chances of disease proliferation, unhygienic conditions cause discomfort to the animals and induce stress (Sejian et al. 2021). Therefore, through extension support and capacity building, farmers need to be enlightened on the need for ensuring a clean and dry shelter environment to keep animals comfortable and well protected from diseases. Ventilation in goat pens was generally good. This is highly encouraging as it ensures efficient heat exchange between the animal and its environment, lowering of relative humidity and accumulation of noxious gases, dust and airborne microbes (Sejian et al. 2021). However, goats were consistently housed with other animals like cattle. This practice induces stress and fosters the spread of diseases. Generally, goats exhibit ancestral predatory fear as well as poor integration with other animal species. Thus, they are often under the most stress from the effects of poor stockmanship.

Regarding flock entries, the reflection that kids born on-farm were a major mode of flock entry corresponds with earlier reports, highlighting that oftentimes farmers obtain livestock replacement stock from their starting stocks (Olwande et al. 2010; Chikumba 2013). Nevertheless, such animal husbandry practices promote inbreeding, ultimately reducing reproductive performance with subsequent declines in flock productivity. Purchases, gifts-in and exchanges were seldom practiced in the current study regardless of their potential for ameliorating the incidences of inbreeding by introducing novel and unlike genotypes to the existing flocks. Financial stumbling blocks in conjunction with the African-centric traditional belief of restocking flocks using existing stocks partly explains why these practices were less common (Henning et al. 2007). Extension services need to be extended to farmers in such societies regarding the impact of inbreeding in reducing reproductive performance. The tendency of using goats for exchanges with other commodities and as tokens of appreciation in various spheres of life evident in the study justify the cultural essence of these animals. Similar previous studies indicated that goats have an immense role in traditional circles, such as being a source of meat during memorial services, dedication of spirit mediums on bucks and religious exorcism of evil spirits on black coated goats (Homann et al. 2007; Mhlanga et al. 2018).

The finding that slaughters were the main cause of flock exits was not unusual. This is in agreement with previous ranking of meat and being a symbol of wealth as the foremost reasons for keeping goats in rural areas (Dossa et al. 2015). Indeed, goats are a crucial source of protein to poor communities regardless of the undervaluation of their contribution to national gross domestic income in African societies (Chiduwa et al. 2008). In Zimbabwe, slaughters are often done during the festive seasons and traditional ceremonies such as paying lobola and religious exorcism of evil spirits, particularly using black coated bucks (Munengwa et al. 2020). The notable proportion of deaths ensuing from parasites and diseases to total exits could be aligned to the poor state of veterinary service support affecting especially this marginalized livestock species (Chiduwa et al. 2008; Munengwa et al. 2020). Also, practices such as housing goats with other animals like cattle and construction of pens directly on the ground may further amplify the spread of infectious diseases between animals. The latter could also be linked with the reports by most of the farmers that they do not regularly remove accumulated manure. Under these situations, urine and faecal material will be poorly drained away from the laying area and creates a conducive environment for growth and multiplication of pathogens. Considering the formidable combination of malnutrition, environmental stress, permanent exposure to parasites and diseases imposed in the natural selection process of native goat breeds in many communal production systems, one would expect goats involved in the study to be intrinsically resistant to diseases (Chiduwa et al. 2008). However, the tendency of using the same breeding stock over several breeding seasons may have resulted in unchecked inbreeding which potentially reduced animal vigor and resistance against diseases.

When compared by environment, the higher incidences of mortalities noted due to parasites and diseases in semi-arid environments than the hot-humid areas seem to suggest that conventional veterinary support, like in many other African societies, is generally confined to environments with favorable climatic conditions for crop farming (Lone et al. 2012). This is supported by the observed pattern where farmers seem to have rely on the use of herbal alternatives for maintaining flock health. As the impact of goat mortalities may be a handicap to sustainable goat productivity in resource-marginalised communities, pharmacological studies are recommended on the potential of forages like *Vachellia* spp. as both nutritional supplements and therapeutic alternatives. Findings may then be cascaded back to communities and effectively guide traditional ethnoveterinary uses in goat primary health care. Also, given the harsh environmental conditions in semi-arid environments, breeding for adaptive traits such as resistance to diseases which are often neglected in conventional breeding objectives for marginalised areas, becomes credible. Another interesting finding is the substantial contribution of predators to total exits, particularly in semi-arid places. This may perhaps be due to limitations of wild prey for predators in these environments as a result of scarcity of feed resources and as such they are forced to prey on domesticated animals. It is recommended that farmers be enlightened on the need to provide appropriate shelter and predator barriers and/or establish effective communication networks between farmers and the parks and wildlife department which is responsible for putting some of the predators like pythons in conservancies.

It was interesting to learn that there were certain farmers who at least follow recommended animal husbandry practices such as implementing proper biosecurity measures and waste disposal. According to Munengwa et al. (2020) disposal patterns such as leaving diseased carcasses, empty antimicrobial containers and expired antimicrobials in situ and/or in water bodies are amongst the chief contributors of the spread of antimicrobial resistance. In clinical terms, antimicrobial resistance reduces treatment efficacy of conventional antimicrobials as well as increasing the overall cost of treatment.

## Conclusions

Goat production is vital for rural livelihoods in communities included in this study, yet its potential is hindered by low reproductive performance due to feed shortages, poor record-keeping, and high parasite loads. Key challenges emanating from these factors include potential inbreeding from closed flock management, inadequate housing affecting animal welfare, and significant disease impact. To improve productivity, we recommend implementing targeted supplementary feeding, establishing community-based breeding programs, strengthening veterinary interventions, and validating the ethnoveterinary practices currently used by farmers.

## Supplementary Information

Below is the link to the electronic supplementary material.

Supplementary Material 1

Supplementary Material 2

## Data Availability

Data generated from the study is not publicly available due to participant’s privacy but are available from the corresponding author upon reasonable request.
