# Supplementary material for: Goat productivity under smallholder farmer management in semi-arid and hot-humid parts of Zimbabwe: a useful step for building resilient goat production systems in marginalized communities
Source: Trop Anim Health Prod. 2026 Jul 31;58(7):467. doi: 10.1007/s11250-026-05215-2 (PMC13427920; doi:10.1007/s11250-026-05215-2)
Supplement: Supplementary file 1 — Supplementary Material 1 [file 11250_2026_5215_MOESM1_ESM.pdf]

Goat productivity under smallholder farmer management in semi-arid and hot-humid parts of Zimbabwe: a useful step for building resilient goat production systems in marginalized communities

Topical Animal Health and Production

Anderson Munengwa[a,d], Emmanuel T. Nyahangare[b], Prosper Jambwa[c], Alban Mugoti[d], Stephen Mandara[d], Melody Dzviti[d], Nation Chikumba[d], Lyndy J. McGaw[a,*]

[a]*Phytomedicine Programme, Department of Paraclinical Sciences, Faculty of Veterinary Science, University of Pretoria, Private Bag X04, Onderstepoort, 0110, Pretoria, South Africa*

[b]*Department of Livestock Sciences, Faculty of Agriculture Environment and Food Systems, University of Zimbabwe, P O Box MP167, Mt Pleasant, Harare, Zimbabwe*

[c]*Department of Veterinary Biosciences, Faculty of Veterinary Science, University of Zimbabwe, P O Box MP167, Mt Pleasant, Harare, Zimbabwe*

[d]*Department of Animal Production Sciences, Faculty of Plant and Animal Science and Technology, Marondera University of Agricultural Sciences and Technology, P O Box 35, Marondera, Zimbabwe*

***Corresponding author**

E-mail address: lyndy.mcgaw@up.ac.za (L.J. McGaw).

Questionnaire number…………

1. **Socio-demographic information:**

**District/ward name/number**	**Gender**		**Age**			**Highest level of education**				**Production experience**		
	Male	Female	18-34	35-64	≥ 65	Non-formal	Primary	Secondary	Tertiary	0-10 years	11-20 years	≥ 20 years

2. **Goat production constraints:**

**How often do you encounter the following production constraints?**	**Always**	**Often**	**Sometimes**	**Seldom**	**Never**
Mortalities due to parasites and diseases					
Poor access to veterinary services					
Feed shortages					
Limited rearing space					
Lack of technical production know-how					
Theft					
Lack of established marketing chain					
Access to markets					
Lack of animal records					
Lack of breeding programs					
Poor access to water					
Predation					

Are there any other production constraints you have encountered before? If YES, specify

………
………
………
………
………
………
………
………
………
………
………
………
………
………
………
………
………
………
………
………
………

3. Goat management practices:

3.1. Goat production system

	**Tethering**	**Extensive grazing**	**Zero grazing**	**Semi intensive**
Which production systems do you adopt for your flock?				

3.2. Goat flock management practices:

**How often do you do the following management practices to your flock?**	**Always**	**Often**	**Sometimes**	**Seldom**	**Never**
Quarantine new additions to the flock for at least 30 days					
Vaccination for diseases					
Keep a closed flock					
Deworming with ethnoveterinary medicines					
Deworming with orthodox drugs					
Ensuring access to clean drinking water					
Provision of commercial feed supplements					
Maintain foot baths at the entrances of pens?					
Burying diseased carcasses					
Burning diseased carcasses					
Leaving diseased carcasses to decay in situ					
Dipping					
Treatment of sick animals with orthodox drugs					
Treatment of sick animals with ethnoveterinary medicines					
Destroy toxic plants					
Consultation of veterinarians for medical advice					

Are there any other management practices you do to maintain the health of your flock? If YES, Specify

……
……
……
……
……
……
……
……
……
……
……
……
……
……
……
……
……

4. Flock compositional characteristics (please record figures):

Flock size	No. of bucks (> 12years)	No. of breeding males (> 12years)	No. of castrates	No. of breeding females (> 12 years)	No. of male kids (< 13 years)	No. of female kids (< 13years)	Total offsprings

5. Selected production indices (please record cases for each productive female:

No. (assign for each female)	No. of offspring surviving to weaning	Litter size	No. of parities per year	No. of females serviced	Age at first service (weeks)	Age at first kidding (weeks)	Gestation length (weeks)

6. Flock entries and exits (please record incidences):

No. of entries						No. of exits						
Kidding	Purchases	Gift-in	Exchanges	Inheritance	Total	Slaughter	Gifts-out	Deaths by diseases	Deaths by injuries	Deaths by predators	Theft	Total

NB. Information captured in this questionnaire will be used solely for research purposes
