# Supplementary material for: Goat productivity under smallholder farmer management in semi-arid and hot-humid parts of Zimbabwe: a useful step for building resilient goat production systems in marginalized communities
Source: Trop Anim Health Prod. 2026 Jul 31;58(7):467. doi: 10.1007/s11250-026-05215-2 (PMC13427920; doi:10.1007/s11250-026-05215-2)
Supplement: Supplementary file 2 — Supplementary Material 2 [file 11250_2026_5215_MOESM2_ESM.pdf]

1. **Flock composition:**

List of abbreviations

DP = Dialogue partner	LOE = Level of education	FZ = Flock size	No. BM = Number of breeding males (> 12 months old)
LS = Litter size/parity	No. PPY = Number of paritie(s)/year	No. FS = Number of females serviced	AFK = Age at first kidding (weeks)
∑. OPY = Total offspring per year	AFS = Age at first service (weeks)	No. BF = Number of breeding females	No. OSW = Number of offspring surviving to weaning
No. MK = Number of male kids	No. FK = Number of female kids	No. Cs = Number of castrates	Av. GL = Average gestation length (months)

Codes applied

**District**	**Gender**	**Age**	**Level of education**	**Goat breeds**
1 = Chikomba (Hot-humid)	1 = Male	1 = 18-34 years old	1 = Non-formal	1 = Indigenous (Mashona/ Matebele)
2 = Murewa (Hot-humid)	2 = Female	2 = 35-64 years old	2 = Primary	2 = Exotic (Boer/ Sainen)
3 = Mwenezi (Semi-arid)		3 = ≥ 65 years old	3 = Secondary	
4 = Gutu (Semi-arid)			4 = Tertiary	

DP	District	Gender	Age	LOE	Breeds	FZ	No. Bucks	No. BM	No. Cs	No. BF	No. MK	No. FK	∑. OPY	No. OSW	LS	No. PPY	No. FS	AFK	Av. GL	AFS
1	1	2	1	1	1	9	2	1	0	3	1	10	11	11	2	2	3	16	4	12
2	1	2	1	1	1	12	1	1	3	3	1	6	7	7	1	2	3	17	5	12
3	1	2	1	1	1	7	1	1	2	4	0	2	2	2	1	1	2	12	4	8
4	1	2	1	1	2	13	0	0	0	5	5	6	11	7	2	2	3	15	4	11
5	1	2	1	1	1	8	0	0	0	4	1	7	8	8	1	2	4	15	4	11
6	1	2	1	2	2	16	1	1	0	6	8	7	15	11	1	2	6	14	5	9
7	1	2	1	2	1	8	0	0	0	2	6	0	6	6	2	2	2	15	4	11
8	1	2	1	2	1	9	2	1	1	4	3	0	3	3	1	1	3	14	5	9
9	1	2	2	2	1	10	0	0	0	4	1	8	9	9	1	2	4	15	4	11
10	1	2	2	3	1	10	0	0	0	3	7	4	11	10	2	2	3	13	5	8
11	1	2	2	3	2	14	1	1	1	3	3	7	10	9	2	2	3	15	4	11
12	1	2	2	3	1	6	1	1	1	4	3	3	6	6	1	2	3	13	4	9
13	1	2	2	3	2	25	2	1	2	7	7	11	18	16	1	2	7	13	4	9
14	1	2	2	3	1	5	0	0	0	2	0	3	3	3	1	1	2	14	4	10
15	1	2	2	3	1	9	1	1	0	3	1	4	5	5	1	2	3	16	5	11
16	1	1	2	3	1	3	0	0	0	1	3	0	3	3	2	2	1	12	4	8
17	1	1	2	3	1	14	0	0	0	3	6	4	10	10	2	2	3	14	5	9
18	1	1	2	3	2	18	0	0	0	5	5	8	13	9	1	2	5	13	5	8
19	1	1	2	3	1	13	0	0	0	5	3	7	10	10	1	2	5	15	4	11
20	1	1	2	3	1	13	1	1	1	4	1	6	7	7	1	2	4	13	5	8
21	1	1	2	3	1	14	2	1	2	8	4	2	6	5	1	1	6	12	4	8
22	1	1	2	3	1	1	1	1	2	3	0	2	2	2	1	1	2	13	4	9
23	1	1	2	3	2	11	1	1	1	5	3	6	9	7	1	2	5	15	5	10
24	1	1	2	3	1	11	1	1	3	5	0	5	5	5	1	1	5	13	5	8
25	1	1	2	3	1	16	1	1	3	5	2	7	9	9	1	2	5	17	5	12
26	1	1	2	3	1	14	2	1	0	3	8	3	11	11	2	2	3	14	4	10
27	1	1	2	3	1	7	1	1	0	4	3	4	7	6	1	2	4	15	4	11
28	1	1	2	3	1	8	1	1	0	3	0	8	8	7	2	2	2	13	4	9
29	1	1	2	3	2	13	1	1	2	4	7	1	8	6	1	2	4	16	4	12
30	1	1	2	3	1	18	2	1	2	5	3	5	8	8	1	2	4	15	4	11
31	1	1	2	3	1	10	1	1	2	4	5	2	8	6	1	2	4	15	4	11

DP	District	Gender	Age	LOE	Breeds	FZ	No. Bucks	No. BM	No. Cs	No. BF	No. MK	No. FK	∑. OPY	No. OSW	LS	No. PPY	No. FS	AFK	Av. GL	AFS
32	1	1	2	3	2	7	1	1	1	4	4	3	7	5	1	2	4	15	4	11
33	1	1	2	3	1	15	1	1	1	4	0	7	7	7	1	2	4	17	5	12
34	1	1	3	3	1	10	1	1	1	4	2	6	8	8	1	2	4	15	4	11
35	1	1	3	3	1	14	1	1	4	4	1	4	5	5	1	1	4	14	5	9
36	1	1	3	3	2	15	1	0	0	6	3	7	10	7	1	2	5	12	4	8
37	1	1	3	3	1	7	1	0	0	4	1	5	6	6	1	1	4	13	4	9
38	1	1	3	3	1	2	1	0	0	3	3	0	3	3	1	1	3	17	4	13
39	1	1	3	3	2	15	1	0	0	8	4	4	8	6	1	1	8	13	4	9
40	1	1	3	3	1	15	1	0	0	7	2	8	10	10	1	1	7	15	4	11
41	1	1	3	3	1	12	2	1	5	4	2	2	4	4	1	1	4	15	4	11
42	1	1	3	3	1	2	1	0	0	1	0	2	2	2	1	2	1	14	6	8
43	1	1	3	4	2	12	2	0	0	5	6	3	9	6	1	2	5	15	5	10
44	1	1	3	4	2	8	1	0	0	4	5	4	9	5	1	2	4	15	5	10
45	1	1	3	4	1	22	1	1	1	6	2	10	12	10	1	2	6	15	5	10
46	1	1	3	4	2	24	1	1	1	8	7	8	15	13	1	2	8	15	4	11
47	1	1	3	4	1	22	1	1	4	7	4	7	11	11	1	2	6	14	5	9
48	1	1	3	4	1	20	1	1	2	9	5	4	9	9	1	1	9	13	5	8
49	1	1	3	4	2	19	2	1	2	9	7	8	15	10	1	2	8	16	5	11
50	1	1	3	4	1	15	2	0	0	6	2	5	7	7	1	1	6	14	4	10
51	2	2	1	1	1	11	2	1	2	6	0	7	7	7	1	1	6	13	4	9
52	2	2	1	1	1	17	1	1	1	5	4	7	11	11	1	2	5	14	4	10
53	2	2	1	1	1	19	1	1	2	8	2	7	9	7	1	1	8	13	4	9
54	2	2	1	1	1	19	1	1	0	8	5	10	15	15	1	2	8	16	4	12
55	2	2	1	1	2	24	0	0	0	8	9	9	18	14	1	2	8	14	4	10
56	2	2	1	2	1	18	0	0	0	6	7	6	13	12	1	2	6	13	5	8
57	2	2	1	2	1	20	0	0	0	7	2	11	13	13	1	2	7	15	5	10
58	2	2	2	2	1	21	0	0	0	7	5	9	14	14	1	2	7	13	5	8
59	2	2	2	3	2	17	0	0	0	7	10	6	16	12	1	2	7	14	4	10
60	2	2	2	3	2	4	0	0	0	3	2	3	5	3	1	2	3	13	4	9
61	2	2	2	3	1	8	0	0	0	3	6	3	9	8	2	2	2	12	4	8
62	2	2	2	3	1	14	0	0	0	3	4	7	11	11	2	2	3	12	4	8

DP	District	Gender	Age	LOE	Breeds	FZ	No. Bucks	No. BM	No. Cs	No. BF	No. MK	No. FK	∑. OPY	No. OSW	LS	No. PPY	No. FS	AFK	Av. GL	AFS
63	2	2	2	3	1	4	0	0	0	3	4	3	7	7	1	2	3	16	4	12
64	2	2	2	3	2	1	0	0	0	3	6	2	8	7	1	2	3	12	4	8
65	2	2	2	3	1	1	0	0	0	2	0	2	2	2	1	1	2	13	4	9
66	2	2	2	3	2	10	0	0	0	4	3	5	8	5	1	2	4	12	4	8
67	2	2	2	3	2	13	0	0	0	4	7	4	11	8	1	2	4	15	5	10
68	2	2	2	3	1	8	0	0	0	4	4	4	8	8	1	2	4	13	5	8
69	2	2	2	3	1	0	0	0	0	2	2	0	2	1	1	1	2	13	4	9
70	2	1	2	3	1	11	0	0	0	6	7	1	8	8	1	1	6	14	5	9
71	2	1	2	3	1	23	0	0	0	9	6	11	17	14	1	2	9	14	4	10
72	2	1	2	3	1	24	0	0	0	9	8	9	17	17	1	2	9	18	5	13
73	2	1	2	3	1	18	1	1	0	6	5	8	13	11	1	2	6	15	4	11
74	2	1	2	3	1	20	1	1	0	6	3	10	13	11	1	2	6	13	4	9
75	2	1	2	3	1	16	1	1	0	7	6	6	12	12	1	2	6	15	4	11
76	2	1	2	3	1	12	1	1	0	7	8	3	11	11	1	1	7	13	4	9
77	2	1	2	3	1	7	1	1	0	3	7	4	11	10	2	2	3	13	4	9
78	2	1	2	3	1	7	1	1	0	3	2	4	6	4	1	2	3	12	4	8
79	2	1	2	3	1	7	2	1	0	2	0	7	7	6	2	2	2	12	4	8
80	2	1	2	3	1	4	0	0	0	2	0	5	5	3	1	2	2	12	4	8
81	2	1	3	3	1	8	0	0	0	2	3	3	6	5	2	2	2	16	4	12
82	2	1	3	3	2	0	0	0	0	1	3	0	3	2	2	2	1	13	4	9
83	2	1	3	3	1	0	0	0	0	1	0	4	4	3	2	2	1	12	4	8
84	2	1	3	3	2	11	0	0	0	7	4	8	12	10	1	2	6	17	4	13
85	2	1	3	3	1	9	0	0	0	6	3	4	7	5	1	1	6	14	4	10
86	2	1	3	3	1	15	0	0	0	6	4	3	7	7	1	1	6	15	4	11
87	2	1	3	3	1	12	0	0	0	5	5	5	10	10	1	2	5	17	4	13
88	2	1	3	3	1	11	0	0	0	6	2	7	9	9	1	1	6	16	5	11
89	2	1	3	3	1	5	1	1	1	5	5	3	8	8	1	2	4	16	4	12
90	2	1	3	3	1	23	1	1	1	6	7	4	11	11	1	2	6	20	5	15
91	2	1	3	3	1	13	1	1	1	6	6	2	8	8	1	1	6	13	5	8
92	2	1	3	3	1	12	1	1	1	3	4	3	7	7	2	2	2	17	5	12

DP	District	Gender	Age	LOE	Breeds	FZ	No. Bucks	No. BM	No. Cs	No. BF	No. MK	No. FK	∑. OPY	No. OSW	LS	No. PPY	No. FS	AFK	Av. GL	AFS
93	2	1	3	3	1	10	1	1	1	3	4	4	8	7	2	2	2	14	4	10
94	2	1	3	4	1	8	1	1	1	4	2	3	5	3	1	1	4	14	4	10
95	2	1	3	4	1	13	1	1	1	4	4	4	8	8	1	2	4	16	4	12
96	2	1	3	4	1	6	1	1	3	3	2	3	5	5	1	2	3	14	5	9
97	2	1	3	4	1	12	1	1	3	3	4	2	6	6	1	2	3	13	5	8
98	2	1	3	4	1	13	1	1	2	3	3	7	10	10	2	2	3	15	5	10
99	2	1	3	4	1	6	1	1	1	1	4	0	4	4	2	2	1	13	4	9
100	2	1	3	4	1	2	1	1	1	1	2	0	2	2	1	2	1	12	4	8
101	3	2	1	1	1	8	0	0	1	1	3	1	4	4	2	2	1	18	5	13
102	3	2	1	1	2	22	3	1	4	4	7	6	13	12	2	2	3	15	4	11
103	3	2	1	1	1	27	2	1	7	5	6	13	19	17	2	2	5	12	4	8
104	3	2	1	1	2	33	2	1	5	5	16	5	21	18	2	2	5	16	5	11
105	3	2	2	1	1	21	2	1	4	6	3	9	12	11	1	2	6	16	4	12
106	3	2	2	1	1	14	1	1	1	6	7	2	9	9	1	2	5	14	4	10
107	3	2	2	2	2	25	1	1	1	9	8	10	18	16	1	2	9	16	6	10
108	3	2	2	2	2	20	1	1	1	8	7	8	15	13	1	2	8	17	5	12
109	3	2	2	2	1	31	1	1	1	8	4	13	17	17	1	2	8	15	5	10
110	3	2	2	2	1	28	1	1	1	10	6	15	21	18	1	2	10	16	4	12
111	3	2	2	2	2	14	2	1	1	5	3	7	10	6	1	2	5	16	4	12
112	3	2	2	3	1	18	3	1	0	8	6	3	9	9	1	1	8	16	4	12
113	3	2	2	3	2	27	3	1	2	8	8	12	20	15	1	2	8	16	4	12
114	3	2	2	3	1	19	3	1	3	3	6	4	10	10	2	2	3	18	4	14
115	3	2	2	3	1	13	2	1	0	3	0	10	10	9	2	2	3	15	4	11
116	3	2	2	3	1	13	0	0	0	6	4	3	7	7	1	1	6	13	4	9
117	3	2	2	3	1	15	1	1	2	4	0	6	6	6	1	1	4	16	4	12
118	3	2	2	3	1	18	1	1	5	6	5	5	10	7	1	2	5	20	5	15
119	3	2	2	3	1	10	1	1	5	2	6	1	7	6	2	2	2	17	4	13
120	3	2	2	3	1	16	1	1	6	6	3	6	9	9	1	2	5	18	4	14
121	3	2	2	3	1	9	0	0	0	6	4	7	11	7	1	2	6	20	6	14
122	3	2	2	3	2	23	0	0	0	9	5	9	14	9	1	1	9	20	5	15

DP	District	Gender	Age	LOE	Breeds	FZ	No. Bucks	No. BM	No. Cs	No. BF	No. MK	No. FK	∑. OPY	No. OSW	LS	No. PPY	No. FS	AFK	Av. GL	AFS
123	3	2	2	3	2	23	0	0	0	10	8	13	21	14	1	2	11	13	5	8
124	3	2	2	3	1	19	2	1	4	7	4	5	9	9	1	1	7	16	5	11
125	3	1	2	3	1	21	2	1	2	8	5	6	11	11	1	1	8	16	5	11
126	3	1	2	3	1	21	2	1	3	8	4	4	8	8	1	1	8	17	5	12
127	3	1	2	3	2	30	2	1	3	9	6	9	15	10	1	2	8	19	5	14
128	3	1	2	3	1	18	1	1	2	3	5	6	11	11	2	2	3	14	5	9
129	3	1	2	3	1	22	1	1	1	9	4	6	10	10	1	1	9	20	4	16
130	3	1	3	3	2	29	1	1	3	9	8	10	18	14	1	2	9	15	4	11
131	3	1	3	3	1	8	1	1	2	2	3	0	3	2	1	2	2	15	4	11
132	3	1	3	3	1	16	1	1	2	3	9	1	10	10	2	2	3	16	4	12
133	3	1	3	3	1	14	1	1	2	3	10	2	12	12	2	2	3	13	5	8
134	3	1	3	3	1	8	1	1	2	1	2	2	4	3	2	2	1	15	5	10
135	3	1	3	3	1	19	1	1	2	6	4	3	7	7	1	1	6	14	5	9
136	3	1	3	3	1	9	1	1	2	1	2	1	3	3	2	2	1	17	5	12
137	3	1	3	3	1	2	1	1	5	1	1	0	1	0	1	1	1	15	5	10
138	3	1	3	3	1	28	2	1	4	8	4	7	11	11	1	1	8	17	5	12
139	3	1	3	3	1	21	2	1	4	8	4	4	8	8	1	1	8	17	4	13
140	3	1	3	3	1	19	0	0	0	8	5	7	12	9	1	1	8	19	5	14
141	3	1	3	3	1	21	0	0	0	9	6	9	15	13	1	2	8	15	6	9
142	3	1	3	3	1	1	0	0	0	1	2	2	4	4	2	2	1	17	4	13
143	3	1	3	3	2	25	0	0	0	10	7	10	17	11	1	2	9	17	5	12
144	3	1	3	3	1	23	0	0	0	10	4	12	16	13	1	2	8	20	4	16
145	3	1	3	3	2	30	0	0	0	12	9	13	22	16	1	2	11	16	4	12
146	3	1	3	3	1	9	0	0	0	5	3	7	10	8	1	2	5	18	4	14
147	3	1	3	4	1	7	0	0	0	5	6	0	6	3	1	1	5	17	5	12
148	3	1	3	4	1	15	0	0	0	4	7	5	12	12	2	2	3	20	5	15
149	3	1	3	4	1	15	0	0	0	3	7	4	11	11	2	2	3	17	4	13
150	3	1	3	4	1	12	0	0	0	3	5	6	11	10	2	2	3	18	5	13
151	4	2	1	1	1	7	1	1	0	3	0	3	3	3	1	1	3	16	6	10
152	4	2	1	1	1	3	0	0	0	2	0	3	3	2	1	2	2	16	5	11

DP	District	Gender	Age	LOE	Breeds	FZ	No. Bucks	No. BM	No. Cs	No. BF	No. MK	No. FK	∑. OPY	No. OSW	LS	No. PPY	No. FS	AFK	Av. GL	AFS
153	4	2	2	1	2	9	0	0	0	2	3	5	8	6	2	2	2	17	4	13
154	4	2	2	2	1	4	0	0	0	2	2	2	4	4	1	2	2	20	4	16
155	4	2	2	2	1	1	0	0	0	2	3	2	5	3	1	2	2	18	6	12
156	4	2	2	2	1	7	0	0	0	2	5	3	8	8	2	2	2	18	4	14
157	4	2	2	2	1	7	0	0	0	5	2	2	4	4	1	1	4	17	4	13
158	4	2	2	2	1	4	0	0	0	4	3	1	4	4	1	1	4	16	4	12
159	4	2	2	3	1	10	0	0	0	4	4	4	8	8	1	2	4	20	4	16
160	4	2	2	3	1	16	0	0	0	7	5	3	8	8	1	1	7	17	5	12
161	4	2	2	3	2	23	1	1	3	7	2	8	10	6	1	2	5	19	4	15
162	4	2	2	3	1	24	1	1	3	7	9	4	13	12	1	2	7	20	5	15
163	4	2	2	3	1	18	1	1	3	3	9	3	12	12	2	2	3	18	4	14
164	4	2	2	3	1	15	0	0	4	4	3	4	7	7	1	2	4	15	5	10
165	4	2	2	3	1	22	0	0	3	9	5	3	8	8	1	1	8	19	5	14
166	4	2	2	3	1	21	0	0	2	9	6	6	12	9	1	1	9	20	5	15
167	4	2	2	3	1	24	1	1	3	11	4	8	12	10	1	1	11	18	4	14
168	4	2	2	3	1	24	1	1	3	9	7	3	10	10	1	1	9	19	4	15
169	4	1	2	3	1	28	1	1	3	5	3	17	20	17	2	2	5	16	5	11
170	4	1	2	3	1	22	1	1	2	9	5	6	11	11	1	1	9	20	6	14
171	4	1	2	3	1	11	1	1	2	6	4	3	7	7	1	1	6	18	5	13
172	4	1	2	3	1	14	1	1	0	6	5	3	8	8	1	1	6	17	5	12
173	4	1	2	3	1	8	0	0	0	6	4	4	8	8	1	1	6	20	6	14
174	4	1	2	3	1	11	1	1	2	3	3	1	4	4	1	1	3	17	4	13
175	4	1	2	3	1	7	1	1	2	1	0	2	2	2	1	2	1	16	4	12
176	4	1	2	3	1	3	0	0	0	1	3	1	4	4	2	2	1	19	4	15
177	4	1	2	3	1	10	1	1	0	4	2	3	5	5	1	1	4	20	4	16
178	4	1	2	3	1	15	0	0	0	4	3	11	14	14	2	2	4	17	4	13
179	4	1	2	3	2	18	0	0	0	7	4	6	10	9	1	1	7	14	4	10
180	4	1	2	3	1	21	0	0	0	7	6	8	14	12	1	2	7	17	4	13
181	4	1	2	3	1	14	1	1	0	8	3	6	9	9	1	1	8	16	4	12
182	4	1	2	3	1	18	1	1	0	8	7	3	10	7	1	1	8	14	5	9

DP	District	Gender	Age	LOE	Breeds	FZ	No. Bucks	No. BM	No. Cs	No. BF	No. MK	No. FK	∑. OPY	No. OSW	LS	No. PPY	No. FS	AFK	Av. GL	AFS
183	4	1	3	3	1	15	1	1	0	8	6	8	14	11	1	2	7	19	5	14
184	4	1	3	3	1	24	1	1	0	12	3	5	8	8	1	1	8	17	5	12
185	4	1	3	3	1	5	1	1	0	3	4	0	4	4	1	1	3	16	5	11
186	4	1	3	3	1	16	0	0	0	6	2	7	9	9	1	1	6	20	5	15
187	4	1	3	3	1	14	0	0	0	3	3	9	12	12	2	2	3	16	5	11
188	4	1	3	3	2	15	0	0	0	9	4	4	8	7	1	1	8	19	6	13
189	4	1	3	3	1	12	0	0	0	7	2	5	7	7	1	1	7	18	4	14
190	4	1	3	3	1	16	0	0	0	8	5	6	11	9	1	1	8	20	4	16
191	4	1	3	3	1	13	0	0	0	3	2	8	10	10	2	2	3	18	6	12
192	4	1	3	3	1	15	1	1	3	8	3	5	8	8	1	1	8	17	4	13
193	4	1	3	3	1	28	1	1	3	11	5	7	12	12	1	1	11	15	5	10
194	4	1	3	3	1	13	1	1	4	4	3	2	5	5	1	1	4	20	4	16
195	4	1	3	4	1	12	1	1	2	7	3	4	7	4	1	1	7	20	4	16
196	4	1	3	4	1	15	1	1	3	6	4	3	7	7	1	1	6	16	4	12
197	4	1	3	4	1	19	2	1	3	6	4	5	9	9	1	1	6	19	4	15
198	4	1	3	4	1	23	2	1	2	9	3	9	12	10	1	1	9	18	5	13
199	4	1	3	4	1	10	2	1	2	3	7	3	10	9	2	2	3	16	4	12
200	4	1	3	4	1	2	2	1	2	1	2	2	4	2	2	2	1	20	5	15

2. Entries and Exits

List of abbreviations

DP = Dialogue partner

LOE = Level of education

Codes applied

District	Gender	Age	Level of education
1 = Chikomba (Hot-humid)	1 = Male	1 = 18-34 years old	1 = Non-formal
2 = Murewa (Hot-humid)	2 = Female	2 = 35-64 years old	2 = Primary
3 = Mwenezi (Semi-arid)		3 = ≥ 65 years old	3 = Secondary
4 = Gutu (Semi-arid)			4 = Tertiary

Sociodemographic factors					Number of entries						Number of exits						
													Mortalities				
DP	Site	Sex	Age	LOE	Kidding	Purchases	Gift-in	Exchanges	Inheritance	Total	Slaughter	Gifts-out	Diseases	Injuries	Predators	Theft	Total
1	1	2	1	1	11	2	1	0	0	14	2	1	3	0	2	2	10
2	1	2	1	1	7	2	1	1	0	11	1	1	2	0	1	1	6
3	1	2	1	1	2	2	0	0	0	4	1	0	1	0	1	1	4
4	1	2	1	1	11	3	0	0	0	14	2	0	2	1	1	0	6
5	1	2	1	1	8	1	2	0	0	11	2	0	2	1	1	1	7
6	1	2	1	2	15	0	0	1	1	17	2	0	4	0	2	0	8
7	1	2	1	2	6	0	1	1	0	8	0	0	2	0	0	0	2
8	1	2	1	2	3	1	0	2	0	6	2	1	1	0	0	0	4
9	1	2	2	2	9	0	1	1	0	11	2	1	2	0	0	0	5
10	1	2	2	3	11	0	2	2	0	15	3	2	3	0	0	0	8
11	1	2	2	3	10	1	0	1	1	13	1	1	2	0	0	0	4
12	1	2	2	3	6	1	1	0	0	8	1	1	3	0	1	2	8
13	1	2	2	3	18	1	0	0	0	19	1	1	2	0	1	0	5
14	1	2	2	3	3	2	1	3	0	9	1	0	4	1	0	0	6
15	1	2	2	3	5	1	1	1	0	8	0	0	2	0	1	0	3
16	1	1	2	3	3	1	1	1	0	6	1	0	3	0	0	0	4
17	1	1	2	3	10	3	0	2	0	15	1	0	2	0	1	0	4
18	1	1	2	3	13	2	0	3	2	20	2	0	4	0	0	1	7
19	1	1	2	3	10	1	1	1	0	13	2	0	2	0	0	1	5
20	1	1	2	3	7	2	1	1	0	11	1	0	2	0	0	1	4
21	1	1	2	3	6	0	0	1	0	7	1	0	1	2	1	0	5
22	1	1	2	3	2	0	1	1	0	4	1	0	4	0	2	2	9
23	1	1	2	3	9	3	0	0	0	12	3	0	2	0	2	1	8
24	1	1	2	3	5	2	1	0	0	8	1	0	4	0	0	1	6
25	1	1	2	3	9	1	0	1	1	12	2	0	2	0	0	1	5
26	1	1	2	3	11	0	1	1	2	15	2	1	3	0	0	0	6
27	1	1	2	3	7	2	1	0	0	10	4	0	2	0	1	1	8
28	1	1	2	3	8	0	0	2	0	10	1	0	4	0	0	1	6
29	1	1	2	3	8	1	0	3	0	12	2	0	4	0	0	0	6

Sociodemographic factors					Number of entries						Number of exits						
													Mortalities				
DP	Site	Sex	Age	LOE	Kidding	Purchases	Gift-in	Exchanges	Inheritance	Total	Slaughter	Gifts-out	Diseases	Injuries	Predators	Theft	Total
30	1	1	2	3	8	1	1	1	0	11	0	0	2	0	0	0	2
31	1	1	2	3	8	0	0	1	0	9	2	0	2	1	1	0	6
32	1	1	2	3	7	2	1	1	0	11	2	2	5	0	1	0	10
33	1	1	2	3	7	3	1	2	3	16	2	0	2	0	1	2	7
34	1	1	3	3	8	3	0	2	0	13	2	0	4	0	0	3	9
35	1	1	3	3	5	2	0	0	0	7	1	0	1	0	0	0	2
36	1	1	3	3	10	1	2	0	0	13	1	0	3	0	1	0	5
37	1	1	3	3	6	0	0	0	0	6	1	0	1	0	2	0	4
38	1	1	3	3	3	1	1	1	1	7	1	0	3	2	3	0	9
39	1	1	3	3	8	0	1	1	1	11	0	0	3	0	1	1	5
40	1	1	3	3	10	1	1	1	1	14	2	0	3	0	1	1	7
41	1	1	3	3	4	2	2	0	0	8	2	0	1	0	3	1	7
42	1	1	3	3	2	3	1	0	0	6	2	0	2	0	1	1	6
43	1	1	3	4	9	1	1	0	0	11	1	0	4	0	0	1	6
44	1	1	3	4	9	0	1	0	0	10	3	0	2	0	0	2	7
45	1	1	3	4	12	1	1	1	3	18	1	0	2	0	1	0	4
46	1	1	3	4	15	0	1	0	3	19	1	0	3	0	1	0	5
47	1	1	3	4	11	0	0	2	4	17	2	2	2	0	1	0	7
48	1	1	3	4	9	2	0	1	2	14	1	2	1	0	0	2	6
49	1	1	3	4	15	3	0	1	0	19	5	1	5	1	1	0	13
50	1	1	3	4	7	0	0	1	5	13	1	0	2	0	3	0	6
51	2	2	1	1	7	1	1	0	0	9	2	0	3	0	2	1	8
52	2	2	1	1	11	0	2	0	1	14	1	0	2	0	1	0	4
53	2	2	1	1	9	0	2	0	1	12	1	0	1	0	2	0	4
54	2	2	1	1	15	0	2	1	1	19	3	1	2	0	2	1	9
55	2	2	1	1	18	2	1	2	3	26	1	1	4	1	2	1	10
56	2	2	1	2	13	3	1	1	1	19	3	1	2	0	1	0	7
57	2	2	1	2	13	2	1	0	1	17	1	0	3	0	0	0	4
58	2	2	2	2	14	0	1	1	2	18	1	0	2	0	0	1	4
59	2	2	2	3	16	0	0	1	0	17	1	2	4	0	0	0	7

Sociodemographic factors					Number of entries						Number of exits						
													Mortalities				
DP	Site	Sex	Age	LOE	Kidding	Purchases	Gift-in	Exchanges	Inheritance	Total	Slaughter	Gifts-out	Diseases	Injuries	Predators	Theft	Total
60	2	2	2	3	5	0	1	2	0	8	3	1	2	0	1	0	7
61	2	2	2	3	9	1	0	0	0	10	2	0	2	0	1	0	5
62	2	2	2	3	11	2	2	0	4	19	2	0	3	1	1	1	8
63	2	2	2	3	7	2	0	1	0	10	3	0	2	1	2	1	9
64	2	2	2	3	8	1	0	1	0	10	3	2	2	1	3	1	12
65	2	2	2	3	2	5	0	0	0	7	3	2	1	0	2	0	8
66	2	2	2	3	8	2	1	1	0	12	1	0	3	0	2	0	6
67	2	2	2	3	11	1	1	1	0	14	2	0	3	0	0	0	5
68	2	2	2	3	8	2	1	1	0	12	5	0	2	0	0	1	8
69	2	2	2	3	2	0	1	0	1	4	2	1	2	0	0	1	6
70	2	1	2	3	8	0	1	0	3	12	1	1	1	1	1	2	7
71	2	1	2	3	17	1	1	2	1	22	2	1	3	0	0	2	8
72	2	1	2	3	17	2	1	0	0	20	2	0	1	0	0	2	5
73	2	1	2	3	13	1	1	0	0	15	1	0	2	0	1	0	4
74	2	1	2	3	13	1	2	0	0	16	1	0	2	0	0	0	3
75	2	1	2	3	12	1	2	1	0	16	3	2	3	0	0	0	8
76	2	1	2	3	11	1	2	0	0	14	2	2	3	0	2	1	10
77	2	1	2	3	11	0	1	0	0	12	2	2	3	1	0	1	9
78	2	1	2	3	6	0	1	2	0	9	2	0	3	0	0	1	6
79	2	1	2	3	7	0	0	1	1	9	4	0	1	0	0	1	6
80	2	1	2	3	5	3	1	1	2	12	1	1	3	0	3	2	10
81	2	1	3	3	6	2	0	1	2	11	2	1	2	0	0	0	5
82	2	1	3	3	3	1	1	0	0	5	1	1	4	0	0	0	6
83	2	1	3	3	4	1	0	0	0	5	4	0	2	0	0	0	6
84	2	1	3	3	12	0	0	0	0	12	3	0	3	1	1	0	8
85	2	1	3	3	7	1	0	0	0	8	2	0	2	0	0	1	5
86	2	1	3	3	7	1	1	0	3	12	2	0	0	0	0	1	3
87	2	1	3	3	10	1	0	2	0	13	1	2	2	0	0	1	6
88	2	1	3	3	9	2	0	0	0	11	1	2	0	0	1	2	6
89	2	1	3	3	8	0	1	0	0	9	3	2	3	0	1	2	11

Sociodemographic factors					Number of entries						Number of exits						
													Mortalities				
DP	Site	Sex	Age	LOE	Kidding	Purchases	Gift-in	Exchanges	Inheritance	Total	Slaughter	Gifts-out	Diseases	Injuries	Predators	Theft	Total
90	2	1	3	3	11	3	1	1	1	17	1	0	0	1	0	0	2
91	2	1	3	3	8	1	0	0	3	12	4	0	1	0	0	2	7
92	2	1	3	3	7	1	2	0	0	10	2	0	0	0	0	1	3
93	2	1	3	3	8	2	1	0	0	11	1	1	3	0	1	0	6
94	2	1	3	4	5	1	0	1	1	8	1	1	2	0	2	0	6
95	2	1	3	4	8	1	2	1	0	12	1	0	0	0	2	2	5
96	2	1	3	4	5	2	2	0	0	9	3	0	3	0	2	2	10
97	2	1	3	4	6	1	0	0	0	7	0	0	0	0	1	1	2
98	2	1	3	4	10	3	2	1	0	16	1	2	4	1	1	0	9
99	2	1	3	4	4	0	1	2	0	7	2	0	0	1	1	0	4
100	2	1	3	4	2	0	2	1	0	5	1	0	3	0	0	2	6
101	3	2	1	1	4	1	2	1	0	8	1	0	0	0	0	1	2
102	3	2	1	1	13	1	0	1	2	17	1	0	1	1	2	1	6
103	3	2	1	1	19	2	0	0	1	22	2	1	2	1	1	2	9
104	3	2	1	1	21	2	0	1	3	27	1	1	0	2	1	1	6
105	3	2	2	1	12	1	1	1	0	15	1	1	1	1	1	1	6
106	3	2	2	1	9	0	1	1	0	11	3	0	0	1	1	0	5
107	3	2	2	2	18	1	0	0	0	19	2	0	2	0	1	0	5
108	3	2	2	2	15	3	1	0	0	19	1	2	2	0	3	1	9
109	3	2	2	2	17	1	2	2	4	26	0	2	0	0	2	1	5
110	3	2	2	2	21	2	1	2	0	26	3	0	3	1	3	0	10
111	3	2	2	2	10	2	1	1	0	14	2	0	2	1	2	1	8
112	3	2	2	3	9	1	0	1	0	11	2	0	0	0	1	1	4
113	3	2	2	3	20	1	1	1	0	23	1	1	0	1	4	2	9
114	3	2	2	3	10	1	1	0	0	12	1	1	0	0	0	0	2
115	3	2	2	3	10	1	0	0	1	12	1	1	1	0	1	0	4
116	3	2	2	3	7	2	1	1	1	12	3	0	0	2	0	0	5
117	3	2	2	3	6	0	2	1	1	10	1	0	0	0	0	1	2
118	3	2	2	3	10	0	2	0	0	12	1	1	3	0	0	1	6
119	3	2	2	3	7	0	2	1	0	10	3	1	1	0	1	2	8

Sociodemographic factors					Number of entries						Number of exits						
													Mortalities				
DP	Site	Sex	Age	LOE	Kidding	Purchases	Gift-in	Exchanges	Inheritance	Total	Slaughter	Gifts-out	Diseases	Injuries	Predators	Theft	Total
120	3	2	2	3	9	1	2	0	0	12	2	1	0	2	1	3	9
121	3	2	2	3	11	2	2	0	0	15	2	0	1	2	3	4	12
122	3	2	2	3	14	1	0	3	3	21	2	0	5	0	0	0	7
123	3	2	2	3	21	3	0	1	0	25	3	1	2	2	3	1	12
124	3	2	2	3	9	1	0	1	0	11	1	0	0	0	2	2	5
125	3	1	2	3	11	1	0	0	0	12	2	0	0	1	0	0	3
126	3	1	2	3	8	1	1	0	0	10	1	0	0	0	1	0	2
127	3	1	2	3	15	2	1	0	4	22	0	0	2	1	2	1	6
128	3	1	2	3	11	3	0	1	0	15	1	1	0	0	0	1	3
129	3	1	2	3	10	2	1	1	1	15	1	1	0	0	0	2	4
130	3	1	3	3	18	2	1	1	0	22	1	0	2	1	1	1	6
131	3	1	3	3	3	0	2	1	0	6	2	0	1	0	0	0	3
132	3	1	3	3	10	1	1	2	0	14	2	0	0	0	1	1	4
133	3	1	3	3	12	0	2	2	0	16	2	2	0	1	1	2	8
134	3	1	3	3	4	0	2	2	0	8	0	0	1	1	1	1	4
135	3	1	3	3	7	1	2	2	2	14	1	0	0	0	2	1	4
136	3	1	3	3	3	1	2	2	0	8	1	0	0	0	0	2	3
137	3	1	3	3	1	0	0	0	0	1	4	1	1	0	0	0	6
138	3	1	3	3	11	2	2	1	2	18	1	0	0	0	3	0	4
139	3	1	3	3	8	2	1	0	0	11	3	0	0	0	0	1	4
140	3	1	3	3	12	1	2	2	0	17	2	0	3	0	0	1	6
141	3	1	3	3	15	0	2	0	0	17	2	1	2	0	0	0	5
142	3	1	3	3	4	0	1	0	0	5	2	0	0	1	1	1	5
143	3	1	3	3	17	1	2	1	1	22	1	0	1	1	4	0	7
144	3	1	3	3	16	1	2	1	0	20	1	0	3	0	2	1	7
145	3	1	3	3	22	2	0	2	0	26	1	0	2	2	3	0	8
146	3	1	3	3	10	1	2	1	0	14	3	2	2	0	2	1	10
147	3	1	3	4	6	0	1	1	0	8	0	0	3	0	3	0	6
148	3	1	3	4	12	1	1	0	4	18	3	0	0	0	4	0	7
149	3	1	3	4	11	1	1	0	0	13	0	0	0	0	0	1	1

Sociodemographic factors					Number of entries						Number of exits						
												Mortalities					
DP	Site	Sex	Age	LOE	Kidding	Purchases	Gift-in	Exchanges	Inheritance	Total	Slaughter	Gifts-out	Diseases	Injuries	Predators	Theft	Total
150	3	1	3	4	11	0	0	1	0	12	2	0	1	0	0	0	3
151	4	2	1	1	3	1	1	2	0	7	0	1	0	1	1	1	4
152	4	2	1	1	3	1	1	1	1	7	1	0	1	0	2	2	6
153	4	2	2	1	8	1	2	2	0	13	1	0	2	1	1	1	6
154	4	2	2	2	4	0	1	1	0	6	1	0	0	1	2	0	4
155	4	2	2	2	5	0	1	1	0	7	2	1	2	1	2	0	8
156	4	2	2	2	8	3	0	0	0	11	3	0	0	0	2	1	6
157	4	2	2	2	4	2	1	0	3	10	3	0	0	0	4	1	8
158	4	2	2	2	4	1	1	2	0	8	3	0	0	0	3	2	8
159	4	2	2	3	8	1	1	1	1	12	1	2	0	0	2	1	6
160	4	2	2	3	8	2	0	1	0	11	1	0	0	0	1	0	2
161	4	2	2	3	10	1	1	3	0	15	0	0	2	0	1	0	3
162	4	2	2	3	13	2	1	0	2	18	2	0	1	1	1	0	5
163	4	2	2	3	12	1	1	1	0	15	1	1	0	0	1	1	4
164	4	2	2	3	7	1	1	1	0	10	1	0	0	0	1	1	3
165	4	2	2	3	8	2	2	3	0	15	1	1	0	1	0	2	5
166	4	2	2	3	12	1	2	1	3	19	2	1	3	1	0	2	9
167	4	2	2	3	12	0	0	1	0	13	2	0	2	0	0	0	4
168	4	2	2	3	10	1	2	2	0	15	2	0	0	0	2	0	4
169	4	1	2	3	20	0	2	0	4	26	1	0	3	0	3	0	7
170	4	1	2	3	11	0	1	3	0	15	3	0	0	1	0	1	5
171	4	1	2	3	7	1	2	0	0	10	4	1	0	1	0	2	8
172	4	1	2	3	8	1	2	1	1	13	1	1	0	0	1	3	6
173	4	1	2	3	8	0	2	1	0	11	4	1	0	0	1	3	9
174	4	1	2	3	4	0	2	1	1	8	1	0	0	1	0	1	3
175	4	1	2	3	2	1	1	3	0	7	2	0	0	2	0	0	4
176	4	1	2	3	4	1	2	2	0	9	1	1	0	2	3	0	7
177	4	1	2	3	5	3	2	2	0	12	1	1	0	1	3	1	7
178	4	1	2	3	14	1	2	1	0	18	1	0	0	1	4	1	7
179	4	1	2	3	10	2	1	0	3	16	2	0	1	1	1	0	5

Sociodemographic factors					Number of entries						Number of exits						
												Mortalities					
DP	Site	Sex	Age	LOE	Kidding	Purchases	Gift-in	Exchanges	Inheritance	Total	Slaughter	Gifts-out	Diseases	Injuries	Predators	Theft	Total
180	4	1	2	3	14	1	2	1	0	18	1	1	2	0	0	0	4
181	4	1	2	3	9	1	1	0	0	11	3	1	0	0	0	2	6
182	4	1	2	3	10	1	1	3	0	15	1	0	3	1	0	1	6
183	4	1	3	3	14	2	1	0	0	17	4	0	3	0	4	0	11
184	4	1	3	3	8	2	2	0	2	14	1	0	0	1	1	0	3
185	4	1	3	3	4	1	1	1	0	7	2	1	0	1	1	1	6
186	4	1	3	3	9	2	2	1	0	14	1	1	0	0	1	1	4
187	4	1	3	3	12	1	1	2	0	16	1	2	0	0	2	0	5
188	4	1	3	3	8	1	1	2	0	12	0	2	1	1	2	0	6
189	4	1	3	3	7	1	0	2	1	11	1	2	0	0	2	1	6
190	4	1	3	3	11	1	1	3	0	16	2	1	2	1	0	2	8
191	4	1	3	3	10	2	1	2	0	15	3	0	0	1	0	1	5
192	4	1	3	3	8	0	1	2	0	11	2	1	0	2	3	0	8
193	4	1	3	3	12	0	0	3	3	18	3	0	0	2	0	0	5
194	4	1	3	3	5	1	2	1	0	9	1	1	0	0	2	1	5
195	4	1	3	4	7	1	0	3	0	11	3	2	3	0	0	1	9
196	4	1	3	4	7	0	1	1	0	9	1	0	0	0	2	1	4
197	4	1	3	4	9	1	1	0	2	13	1	2	0	0	0	2	5
198	4	1	3	4	12	0	1	0	2	15	1	0	2	1	1	0	5
199	4	1	3	4	10	0	0	1	0	11	3	2	1	1	1	0	8
200	4	1	3	4	4	0	2	0	2	8	4	2	2	0	1	2	11

3. Goat production constraints

List of abbreviations

DP = dialogue partner

Site	Gender	Age	Level of education	Production constraints (both code and score)
1 = Chikomba (Hot-humid)	1 = Male	1 = 18-34 years old	1 = Non-formal	1 = Never
2 = Murewa (Hot-humid)	2 = Female	2 = 35-64 years old	2 = Primary	2 = Seldom
3 = Mwenezi (Semi-arid)		3 = ≥ 65 years old	3 = Secondary	3 = Sometimes
4 = Gutu (Semi-arid)			4 = Tertiary	4 = Often
				5 = Always

NB. Scores from 1 to 5 for production constraints indicate order of importance

DP	Site	Sex	Age	Level of education	Diseases	Clean water	Veterinary services	Technical know-how	Theft	Market access	Breeding males	Predators	Rearing space	Feed shortage
1	1	2	1	1	5	1	5	2	4	1	1	2	1	1
2	1	2	1	1	5	1	5	2	5	1	1	2	1	1
3	1	2	1	1	2	1	5	2	5	1	1	3	1	1
4	1	2	1	1	4	1	4	3	1	1	5	2	1	1
5	1	2	1	1	5	1	4	3	5	1	3	2	1	1
6	1	2	1	2	5	1	3	1	1	1	1	4	1	2
7	1	2	1	2	2	1	3	2	1	1	3	1	1	2
8	1	2	1	2	5	1	3	3	1	1	1	1	5	2
9	1	2	2	2	5	1	2	2	1	1	3	1	5	1
10	1	2	2	3	4	1	2	2	1	1	4	1	5	1
11	1	2	2	3	1	2	3	3	1	2	1	1	1	1
12	1	2	2	3	3	2	3	3	4	3	1	2	1	1
13	1	2	2	3	5	1	2	4	1	3	1	2	1	1
14	1	2	2	3	3	2	1	3	1	3	5	1	1	1
15	1	2	2	3	5	4	3	2	2	3	2	1	1	1
16	1	1	2	3	2	4	3	5	1	3	5	1	1	1
17	1	1	2	3	5	5	4	5	1	3	4	5	1	3
18	1	1	2	3	4	1	4	5	2	2	5	1	1	1
19	1	1	2	3	1	1	4	4	2	2	5	1	1	1
20	1	1	2	3	3	1	3	4	5	2	1	1	1	1
21	1	1	2	3	4	2	3	2	1	2	2	2	1	1
22	1	1	2	3	5	4	1	3	4	2	2	4	1	1
23	1	1	2	3	4	4	5	4	5	2	1	4	1	1
24	1	1	2	3	4	1	5	4	2	2	1	1	1	3
25	1	1	2	3	4	1	4	3	5	1	1	1	5	1
26	1	1	2	3	5	3	5	2	1	1	1	1	1	1
27	1	1	2	3	1	1	3	3	2	1	1	5	1	1
28	1	1	2	3	3	3	3	4	2	1	1	1	1	1
29	1	1	2	3	5	1	2	4	2	1	1	1	1	2

DP	Site	Sex	Age	Level of education	Diseases	Clean water	Veterinary services	Technical know-how	Theft	Market access	Breeding males	Predators	Rearing space	Feed shortage
30	1	1	2	3	5	1	2	4	1	1	1	1	1	2
31	1	1	2	3	3	3	2	5	1	1	1	3	1	2
32	1	1	2	3	5	1	4	4	1	1	1	3	1	1
33	1	1	2	3	5	1	4	3	4	1	1	2	1	1
34	1	1	3	3	5	1	4	4	3	1	2	1	1	1
35	1	1	3	3	2	1	1	5	1	1	2	1	1	3
36	1	1	3	3	2	1	1	4	1	1	4	2	1	1
37	1	1	3	3	1	1	5	3	1	5	4	4	1	1
38	1	1	3	3	5	2	4	4	1	2	5	3	1	1
39	1	1	3	3	2	3	4	1	2	2	5	5	1	1
40	1	1	3	3	5	3	4	1	3	2	5	5	5	1
41	1	1	3	3	1	1	4	2	2	4	1	3	1	1
42	1	1	3	3	1	1	3	1	4	4	4	5	1	2
43	1	1	3	4	5	3	3	2	5	4	3	1	1	1
44	1	1	3	4	3	1	5	3	4	5	3	1	1	1
45	1	1	3	4	3	5	4	3	1	5	1	5	1	1
46	1	1	3	4	5	1	2	3	1	5	1	5	1	3
47	1	1	3	4	5	3	3	3	1	5	1	5	1	3
48	1	1	3	4	5	4	4	3	4	1	1	1	1	1
49	1	1	3	4	5	2	5	2	1	1	1	2	1	1
50	1	1	3	4	5	4	4	2	1	1	4	3	1	1
51	2	2	1	1	4	2	2	2	5	1	2	4	1	1
52	2	2	1	1	1	2	2	2	2	1	3	2	1	1
53	2	2	1	1	5	2	2	1	1	1	3	4	1	1
54	2	2	1	1	5	4	4	1	5	1	3	4	1	1
55	2	2	1	1	5	1	5	2	5	1	4	2	1	1
56	2	2	1	2	2	2	3	2	1	1	4	2	1	1
57	2	2	1	2	2	2	2	3	1	3	5	1	1	1
58	2	2	2	2	5	2	4	4	5	3	5	1	1	2
59	2	2	2	3	5	4	4	3	1	2	5	1	1	1

DP	Site	Sex	Age	Level of education	Diseases	Clean water	Veterinary services	Technical know-how	Theft	Market access	Breeding males	Predators	Rearing space	Feed shortage
60	2	2	2	3	5	3	1	2	1	1	4	4	1	1
61	2	2	2	3	1	3	4	3	1	1	4	2	1	1
62	2	2	2	3	2	3	3	4	2	2	4	3	1	1
63	2	2	2	3	5	2	4	3	5	2	5	2	1	1
64	2	2	2	3	5	2	1	2	2	2	5	3	1	1
65	2	2	2	3	5	4	4	3	1	2	5	4	1	1
66	2	2	2	3	5	2	4	1	1	1	3	2	1	1
67	2	2	2	3	1	4	4	2	1	1	4	1	5	1
68	2	2	2	3	5	3	5	3	2	1	4	1	1	1
69	2	2	2	3	1	2	3	3	5	1	4	1	1	1
70	2	1	2	3	1	2	3	2	4	1	4	5	1	2
71	2	1	2	3	5	4	4	2	4	1	5	1	1	1
72	2	1	2	3	5	3	3	1	4	1	5	1	1	1
73	2	1	2	3	5	4	3	2	1	4	1	3	1	1
74	2	1	2	3	5	2	1	3	1	4	1	1	1	3
75	2	1	2	3	1	4	4	2	1	5	1	1	1	3
76	2	1	2	3	4	3	4	2	5	5	1	4	1	3
77	2	1	2	3	5	2	5	1	5	1	1	1	5	1
78	2	1	2	3	5	4	3	2	5	1	1	1	5	1
79	2	1	2	3	4	3	1	2	5	1	1	1	1	1
80	2	1	2	3	1	3	4	3	4	2	4	3	1	1
81	2	1	3	3	3	3	5	4	1	2	5	1	1	1
82	2	1	3	3	5	2	4	4	1	1	5	1	1	2
83	2	1	3	3	5	4	3	3	1	1	5	1	1	2
84	2	1	3	3	2	3	3	2	1	1	3	5	1	1
85	2	1	3	3	5	2	4	3	5	1	5	1	5	1
86	2	1	3	3	5	2	5	4	5	1	4	1	1	1
87	2	1	3	3	2	2	4	4	5	2	4	1	1	1
88	2	1	3	3	5	3	4	3	4	2	4	2	1	1
89	2	1	3	3	5	1	5	2	4	2	1	2	1	1

DP	Site	Sex	Age	Level of education	Diseases	Clean water	Veterinary services	Technical know-how	Theft	Market access	Breeding males	Predators	Rearing space	Feed shortage
90	2	1	3	3	1	5	3	3	3	2	1	1	1	1
91	2	1	3	3	2	4	5	3	4	2	1	1	1	1
92	2	1	3	3	5	2	3	4	3	2	1	1	1	1
93	2	1	3	3	2	4	4	3	1	5	1	2	1	1
94	2	1	3	4	5	3	5	3	1	5	1	2	1	1
95	2	1	3	4	5	5	3	4	4	1	2	2	1	1
96	2	1	3	4	5	3	2	4	4	1	2	2	1	1
97	2	1	3	4	5	2	2	3	3	1	3	3	1	1
98	2	1	3	4	5	3	5	2	3	1	3	2	1	1
99	2	1	3	4	5	3	5	2	3	1	3	5	1	2
100	2	1	3	4	5	4	5	1	4	1	3	1	1	1
101	3	2	1	1	1	4	1	3	3	2	5	1	1	1
102	3	2	1	1	5	2	5	3	3	5	1	4	1	1
103	3	2	1	1	4	5	5	3	4	4	2	2	1	1
104	3	2	1	1	1	2	5	1	3	5	2	2	1	1
105	3	2	2	1	5	5	5	3	5	4	2	4	5	1
106	3	2	2	1	4	2	5	2	1	5	2	2	5	3
107	3	2	2	2	1	5	4	2	1	2	1	5	1	2
108	3	2	2	2	5	4	4	2	5	2	1	3	1	2
109	3	2	2	2	4	4	2	1	5	2	1	4	1	2
110	3	2	2	2	5	5	3	2	3	2	1	3	1	2
111	3	2	2	2	3	2	4	3	5	2	1	2	1	1
112	3	2	2	3	4	5	5	1	5	2	1	5	1	1
113	3	2	2	3	1	1	5	1	4	1	1	2	1	1
114	3	2	2	3	4	5	4	1	2	1	1	1	1	3
115	3	2	2	3	2	4	3	1	1	1	1	5	1	1
116	3	2	2	3	5	2	4	4	3	1	5	1	1	1
117	3	2	2	3	4	4	4	4	5	1	2	1	1	1
118	3	2	2	3	5	4	5	5	5	1	2	1	1	1
119	3	2	2	3	4	5	5	5	4	3	1	5	1	2

DP	Site	Sex	Age	Level of education	Diseases	Clean water	Veterinary services	Technical know-how	Theft	Market access	Breeding males	Predators	Rearing space	Feed shortage
120	3	2	2	3	4	2	5	5	3	1	1	5	1	2
121	3	2	2	3	3	2	5	5	2	1	4	3	1	2
122	3	2	2	3	5	5	5	5	1	3	3	1	1	2
123	3	2	2	3	1	2	5	4	3	3	4	3	1	2
124	3	2	2	3	5	4	5	3	4	1	1	4	1	1
125	3	1	2	3	5	5	5	2	1	1	1	1	1	1
126	3	1	2	3	5	4	3	2	2	1	1	5	1	1
127	3	1	2	3	3	5	2	3	3	1	1	4	1	1
128	3	1	2	3	1	4	3	4	5	1	1	1	1	3
129	3	1	2	3	4	4	4	5	4	2	1	1	1	3
130	3	1	3	3	5	5	4	5	5	2	2	5	1	3
131	3	1	3	3	5	4	4	4	1	2	2	2	1	2
132	3	1	3	3	4	5	4	3	3	2	2	5	1	1
133	3	1	3	3	4	1	3	4	4	1	1	4	1	1
134	3	1	3	3	5	5	4	4	5	1	1	5	1	1
135	3	1	3	3	4	4	4	4	5	1	1	4	1	1
136	3	1	3	3	4	4	4	5	4	1	1	2	1	1
137	3	1	3	3	5	4	4	4	2	2	1	1	1	1
138	3	1	3	3	4	4	3	3	1	1	1	3	1	2
139	3	1	3	3	4	4	5	2	3	2	1	1	1	2
140	3	1	3	3	5	4	4	3	4	1	5	1	1	1
141	3	1	3	3	5	1	1	4	2	2	5	1	1	1
142	3	1	3	3	5	1	4	4	5	4	5	5	1	1
143	3	1	3	3	4	5	4	5	1	5	5	2	1	1
144	3	1	3	3	4	4	1	4	5	2	4	4	1	1
145	3	1	3	3	1	2	1	4	1	1	3	3	1	3
146	3	1	3	3	5	4	4	3	5	1	3	4	1	2
147	3	1	3	4	3	4	5	2	1	4	4	3	1	2
148	3	1	3	4	4	1	4	3	1	1	4	2	1	2
149	3	1	3	4	5	4	2	4	5	1	5	1	1	3

DP	Site	Sex	Age	Level of education	Diseases	Clean water	Veterinary services	Technical know-how	Theft	Market access	Breeding males	Predators	Rearing space	Feed shortage
150	3	1	3	4	3	4	3	5	1	1	5	1	1	2
151	4	2	1	1	5	2	4	5	4	5	1	5	1	2
152	4	2	1	1	5	3	4	5	4	3	5	4	1	2
153	4	2	2	1	5	5	4	5	4	3	4	5	1	2
154	4	2	2	2	4	3	4	2	1	3	4	4	5	2
155	4	2	2	2	1	3	4	3	1	2	4	4	1	3
156	4	2	2	2	4	4	4	4	4	2	3	4	1	3
157	4	2	2	2	4	4	5	5	5	1	3	2	1	2
158	4	2	2	2	4	1	5	5	4	1	4	3	1	2
159	4	2	2	3	5	4	5	4	5	1	2	4	1	2
160	4	2	2	3	5	4	5	3	2	1	3	4	1	1
161	4	2	2	3	1	5	3	2	3	1	2	3	1	3
162	4	2	2	3	5	5	4	1	1	1	1	5	1	3
163	4	2	2	3	4	4	5	2	4	5	1	5	1	1
164	4	2	2	3	4	4	5	3	4	4	5	5	1	1
165	4	2	2	3	4	1	3	4	4	1	4	1	1	1
166	4	2	2	3	5	1	4	3	4	1	5	1	1	1
167	4	2	2	3	5	4	2	3	1	1	1	1	1	2
168	4	2	2	3	4	3	2	2	1	1	1	4	1	1
169	4	1	2	3	5	4	4	2	1	2	1	3	1	1
170	4	1	2	3	4	5	5	3	5	1	1	1	1	1
171	4	1	2	3	4	2	1	4	4	1	2	1	1	2
172	4	1	2	3	4	4	5	5	3	1	1	4	1	1
173	4	1	2	3	4	4	1	5	3	1	4	5	1	1
174	4	1	2	3	4	1	5	3	5	1	1	1	5	2
175	4	1	2	3	4	2	1	1	1	1	1	1	1	3
176	4	1	2	3	4	2	1	4	1	4	4	3	1	3
177	4	1	2	3	4	5	5	4	5	5	1	3	1	2
178	4	1	2	3	4	5	5	4	5	5	4	2	1	3
179	4	1	2	3	3	1	4	1	1	5	5	3	1	2

DP	Site	Sex	Age	Level of education	Diseases	Clean water	Veterinary services	Technical know-how	Theft	Market access	Breeding males	Predators	Rearing space	Feed shortage
180	4	1	2	3	5	5	3	4	1	1	5	1	1	3
181	4	1	2	3	5	4	4	4	4	1	1	1	5	1
182	4	1	2	3	4	5	5	5	5	1	1	1	1	1
183	4	1	3	3	1	5	4	1	1	1	1	2	1	4
184	4	1	3	3	1	1	3	5	1	1	2	3	1	1
185	4	1	3	3	5	5	5	5	2	1	3	3	1	1
186	4	1	3	3	5	4	5	5	2	2	3	3	1	1
187	4	1	3	3	5	5	4	5	1	1	3	4	1	1
188	4	1	3	3	4	1	5	5	1	1	3	3	1	2
189	4	1	3	3	4	4	4	5	3	1	4	4	1	4
190	4	1	3	3	5	5	5	4	4	2	5	1	1	1
191	4	1	3	3	4	4	4	5	5	2	5	1	1	1
192	4	1	3	3	4	2	2	3	1	2	3	3	1	1
193	4	1	3	3	4	2	3	5	1	3	3	1	1	1
194	4	1	3	3	4	4	5	5	2	1	3	4	1	1
195	4	1	3	4	5	3	5	5	2	1	3	2	1	1
196	4	1	3	4	4	4	3	5	5	1	1	4	1	2
197	4	1	3	4	4	5	4	3	4	1	1	3	1	3
198	4	1	3	4	2	5	5	4	1	1	1	5	1	1
199	4	1	3	4	5	1	4	4	1	1	1	3	1	1
200	4	1	3	4	1	4	5	3	4	4	1	4	1	1

4. Mortalities by disease category

List of abbreviations

DP = dialogue partner

Codes applied

Site	Gender	Age	Level of education
1 = Chikomba (Hot-humid)	1 = Male	1 = 18-34 years old	1 = Non-formal
2 = Murewa (Hot-humid)	2 = Female	2 = 35-64 years old	2 = Primary
3 = Mwenezi (Semi-arid)		3 = ≥ 65 years old	3 = Secondary
4 = Gutu (Semi-arid)			4 = Tertiary

NB. Values in each disease category are recorded cases for one complete year

DP	Site	Sex	Age	Level of education	Infectious and parasitic diseases	Respiratory diseases	Pre- and post-partum reproductive diseases	Digestive diseases
1	1	2	1	1	1	1	1	0
2	1	2	1	1	1	0	0	1
3	1	2	1	1	1	0	0	0
4	1	2	1	1	0	0	1	1
5	1	2	1	1	1	0	1	0
6	1	2	1	2	2	1	0	1
7	1	2	1	2	0	0	1	1
8	1	2	1	2	1	0	0	0
9	1	2	2	2	0	1	0	1
10	1	2	2	3	2	0	0	1
11	1	2	2	3	2	0	0	0
12	1	2	2	3	1	1	1	0
13	1	2	2	3	1	0	0	1
14	1	2	2	3	1	1	1	1
15	1	2	2	3	1	0	0	1
16	1	1	2	3	1	0	1	1
17	1	1	2	3	0	0	1	1
18	1	1	2	3	3	0	0	1
19	1	1	2	3	0	0	1	1
20	1	1	2	3	2	0	0	0
21	1	1	2	3	1	0	0	0
22	1	1	2	3	2	1	0	1
23	1	1	2	3	0	0	0	2
24	1	1	2	3	2	0	0	2
25	1	1	2	3	2	0	0	0
26	1	1	2	3	2	0	1	0
27	1	1	2	3	1	0	0	1
28	1	1	2	3	2	1	0	1
29	1	1	2	3	3	0	0	1

DP	Site	Sex	Age	Level of education	Infectious and parasitic diseases	Respiratory diseases	Pre- and post-partum reproductive diseases	Digestive diseases
30	1	1	2	3	1	0	0	1
31	1	1	2	3	1	0	1	0
32	1	1	2	3	3	1	1	0
33	1	1	2	3	1	0	0	1
34	1	1	3	3	0	0	2	2
35	1	1	3	3	0	0	1	0
36	1	1	3	3	1	2	0	0
37	1	1	3	3	0	1	0	0
38	1	1	3	3	2	0	1	0
39	1	1	3	3	1	0	0	2
40	1	1	3	3	2	1	0	0
41	1	1	3	3	0	0	1	0
42	1	1	3	3	0	0	0	2
43	1	1	3	4	1	3	0	0
44	1	1	3	4	2	0	0	0
45	1	1	3	4	1	0	0	1
46	1	1	3	4	2	0	0	1
47	1	1	3	4	0	0	2	0
48	1	1	3	4	0	0	0	1
49	1	1	3	4	2	1	2	0
50	1	1	3	4	0	2	0	0
51	2	2	1	1	1	0	0	2
52	2	2	1	1	0	2	0	0
53	2	2	1	1	1	0	0	0
54	2	2	1	1	2	0	0	0
55	2	2	1	1	1	3	0	0
56	2	2	1	2	2	0	0	0
57	2	2	1	2	1	0	0	2
58	2	2	2	2	0	0	2	0
59	2	2	2	3	2	2	0	0

DP	Site	Sex	Age	Level of education	Infectious and parasitic diseases	Respiratory diseases	Pre- and post-partum reproductive diseases	Digestive diseases
60	2	2	2	3	0	0	2	0
61	2	2	2	3	2	0	0	0
62	2	2	2	3	0	1	1	1
63	2	2	2	3	2	0	0	0
64	2	2	2	3	1	0	1	0
65	2	2	2	3	0	0	1	0
66	2	2	2	3	0	2	1	0
67	2	2	2	3	0	1	0	2
68	2	2	2	3	2	0	0	0
69	2	2	2	3	1	1	0	0
70	2	1	2	3	0	0	1	0
71	2	1	2	3	2	1	0	0
72	2	1	2	3	1	0	0	0
73	2	1	2	3	0	0	0	2
74	2	1	2	3	0	1	1	0
75	2	1	2	3	0	0	2	1
76	2	1	2	3	3	0	0	0
77	2	1	2	3	0	0	2	1
78	2	1	2	3	2	1	0	0
79	2	1	2	3	0	0	1	0
80	2	1	2	3	2	0	0	1
81	2	1	3	3	0	0	0	2
82	2	1	3	3	1	2	0	1
83	2	1	3	3	0	0	2	0
84	2	1	3	3	0	0	0	3
85	2	1	3	3	0	0	2	0
86	2	1	3	3	0	0	0	0
87	2	1	3	3	2	0	0	0
88	2	1	3	3	0	0	0	0
89	2	1	3	3	1	2	0	0

DP	Site	Sex	Age	Level of education	Infectious and parasitic diseases	Respiratory diseases	Pre- and post-partum reproductive diseases	Digestive diseases
90	2	1	3	3	0	0	0	0
91	2	1	3	3	1	0	0	0
92	2	1	3	3	0	0	0	0
93	2	1	3	3	2	1	0	0
94	2	1	3	4	0	0	1	1
95	2	1	3	4	0	0	0	0
96	2	1	3	4	1	1	1	0
97	2	1	3	4	0	0	0	0
98	2	1	3	4	0	0	2	2
99	2	1	3	4	0	0	0	0
100	2	1	3	4	1	1	1	0
101	3	2	1	1	0	0	0	0
102	3	2	1	1	1	0	0	0
103	3	2	1	1	2	0	0	0
104	3	2	1	1	0	0	0	0
105	3	2	2	1	0	0	0	1
106	3	2	2	1	0	0	0	0
107	3	2	2	2	1	0	1	0
108	3	2	2	2	1	1	0	0
109	3	2	2	2	0	0	0	0
110	3	2	2	2	1	0	1	1
111	3	2	2	2	0	2	0	0
112	3	2	2	3	0	0	0	0
113	3	2	2	3	0	0	0	0
114	3	2	2	3	0	0	0	0
115	3	2	2	3	1	0	0	0
116	3	2	2	3	0	0	0	0
117	3	2	2	3	0	0	0	0
118	3	2	2	3	0	0	0	2
119	3	2	2	3	0	1	0	0

DP	Site	Sex	Age	Level of education	Infectious and parasitic diseases	Respiratory diseases	Pre- and post-partum reproductive diseases	Digestive diseases
120	3	2	2	3	0	0	0	0
121	3	2	2	3	1	0	0	0
122	3	2	2	3	0	0	3	2
123	3	2	2	3	1	1	0	0
124	3	2	2	3	0	0	0	0
125	3	1	2	3	0	0	0	0
126	3	1	2	3	0	0	0	0
127	3	1	2	3	1	0	0	1
128	3	1	2	3	0	0	0	0
129	3	1	2	3	0	0	0	0
130	3	1	3	3	0	0	1	1
131	3	1	3	3	1	0	0	0
132	3	1	3	3	0	0	0	0
133	3	1	3	3	0	0	0	0
134	3	1	3	3	0	1	0	0
135	3	1	3	3	0	0	0	0
136	3	1	3	3	0	0	0	0
137	3	1	3	3	0	0	1	0
138	3	1	3	3	0	0	0	0
139	3	1	3	3	0	0	0	0
140	3	1	3	3	2	0	0	1
141	3	1	3	3	1	1	0	0
142	3	1	3	3	0	0	0	0
143	3	1	3	3	0	0	0	1
144	3	1	3	3	2	0	1	0
145	3	1	3	3	0	0	1	1
146	3	1	3	3	0	2	0	0
147	3	1	3	4	1	0	2	0
148	3	1	3	4	0	0	0	0
149	3	1	3	4	0	0	0	0

DP	Site	Sex	Age	Level of education	Infectious and parasitic diseases	Respiratory diseases	Pre- and post-partum reproductive diseases	Digestive diseases
150	3	1	3	4	1	0	0	0
151	4	2	1	1	0	0	0	0
152	4	2	1	1	0	0	0	1
153	4	2	2	1	1	1	0	0
154	4	2	2	2	0	0	0	0
155	4	2	2	2	1	0	0	1
156	4	2	2	2	0	0	0	0
157	4	2	2	2	0	0	0	0
158	4	2	2	2	0	0	0	0
159	4	2	2	3	0	0	0	0
160	4	2	2	3	0	0	0	0
161	4	2	2	3	0	0	0	2
162	4	2	2	3	1	0	0	0
163	4	2	2	3	0	0	0	0
164	4	2	2	3	0	0	0	0
165	4	2	2	3	0	0	0	0
166	4	2	2	3	1	2	0	0
167	4	2	2	3	1	0	1	0
168	4	2	2	3	0	0	0	0
169	4	1	2	3	0	1	1	1
170	4	1	2	3	0	0	0	0
171	4	1	2	3	0	0	0	0
172	4	1	2	3	0	0	0	0
173	4	1	2	3	0	0	0	0
174	4	1	2	3	0	0	0	0
175	4	1	2	3	0	0	0	0
176	4	1	2	3	0	0	0	0
177	4	1	2	3	0	0	0	0
178	4	1	2	3	0	0	0	0
179	4	1	2	3	0	0	0	1

DP	Site	Sex	Age	Level of education	Infectious and parasitic diseases	Respiratory diseases	Pre- and post-partum reproductive diseases	Digestive diseases
180	4	1	2	3	2	0	0	0
181	4	1	2	3	0	0	0	0
182	4	1	2	3	1	2	0	0
183	4	1	3	3	0	0	1	2
184	4	1	3	3	0	0	0	0
185	4	1	3	3	0	0	0	0
186	4	1	3	3	0	0	0	0
187	4	1	3	3	0	0	0	0
188	4	1	3	3	1	0	0	0
189	4	1	3	3	0	0	0	0
190	4	1	3	3	0	1	1	0
191	4	1	3	3	0	0	0	0
192	4	1	3	3	0	0	0	0
193	4	1	3	3	0	0	0	0
194	4	1	3	3	0	0	0	0
195	4	1	3	4	1	1	1	0
196	4	1	3	4	0	0	0	0
197	4	1	3	4	0	0	0	0
198	4	1	3	4	0	0	0	2
199	4	1	3	4	0	0	1	0
200	4	1	3	4	0	2	0	0

5. Goat management practices

List of abbreviations

DP = dialogue partner

QSA = quarantine sick animals

DOD = deworming with orthodox drugs

BDC = burying diseased carcasses

MSCSV = maintenance of shelter sanitary conditions

DTP = destruction of toxic plants

PS = production system

WFV = whole flock vaccination

DE = deworming with ethnoveterinary medicines

LDCIS = leaving diseased carcasses in situ

TSAOD = use of orthodox drugs for ailments

TSAED = use of ethnoveterinary medicines for ailments

PE = production experience

KCF = keeping closed flock

ACW = access to clean water

BuDC = burning diseased carcasses

Codes applied

Site	Gender	Age	Level of education	Management practices (both code and score)
1 = Chikomba (Hot-humid)	1 = Male	1 = 18-34 years old	1 = Non-formal	1 = Never
2 = Murewa (Hot-humid)	2 = Female	2 = 35-64 years old	2 = Primary	2 = Seldom
3 = Mwenezi (Semi-arid)		3 = ≥ 65 years old	3 = Secondary	3 = Sometimes
4 = Gutu (Semi-arid)			4 = Tertiary	4 = Often
**Production experience**				5 = Always
1 = 0-10 years				
2 = 11-20 years				
3 = > 20 years				

NB. Scores from 1 to 5 for management practices indicate order of importance

DP	Site	Sex	Age	Level of education	PS	PE	QSA	WFV	KCF	DOD	DE	ACW	BDC	BuDC	LDCIS	MSCS	TSAOD	TSAED	DTP
1	1	2	1	1	1	1	1	1	1	3	2	4	4	1	1	1	1	2	1
2	1	2	1	1	1	1	1	1	2	1	2	4	5	1	1	1	1	2	1
3	1	2	1	1	1	1	1	1	1	1	2	5	5	1	1	1	1	2	2
4	1	2	1	1	1	1	1	1	3	3	2	5	5	2	1	4	1	2	1
5	1	2	1	1	1	1	1	1	1	2	3	5	5	2	1	1	2	3	2
6	1	2	1	2	2	1	2	1	1	1	2	4	5	1	2	1	3	2	2
7	1	2	1	2	1	1	1	1	2	1	2	5	4	1	1	1	3	2	2
8	1	2	1	2	1	1	1	1	1	2	3	5	4	1	1	4	4	3	1
9	1	2	2	2	2	2	1	1	1	1	3	4	4	3	1	4	4	5	4
10	1	2	2	3	2	2	1	1	3	1	4	5	3	3	1	4	4	4	3
11	1	2	2	3	2	2	1	1	2	2	4	5	3	1	2	1	3	5	3
12	1	2	2	3	1	2	2	1	1	1	4	4	3	1	1	1	3	5	4
13	1	2	2	3	3	2	3	2	2	3	3	4	3	2	1	3	4	5	2
14	1	2	2	3	1	2	1	1	3	1	4	4	3	1	1	3	4	4	2
15	1	2	2	3	1	2	1	1	2	1	3	5	5	2	1	1	3	3	4
16	1	1	2	3	1	2	1	1	1	3	3	5	5	1	1	1	3	3	2
17	1	1	2	3	2	3	2	1	2	1	5	5	5	2	3	2	3	5	5
18	1	1	2	3	4	3	2	1	5	3	5	5	5	1	1	1	4	5	5
19	1	1	2	3	4	3	2	1	1	1	4	4	5	3	1	1	4	5	5
20	1	1	2	3	4	3	1	1	2	2	5	4	4	1	1	1	4	5	5
21	1	1	2	3	4	3	1	1	1	1	4	5	4	4	1	1	3	5	5
22	1	1	2	3	1	3	1	1	2	1	4	5	4	1	1	5	3	5	5
23	1	1	2	3	2	3	2	1	1	2	5	4	4	3	1	1	3	5	4
24	1	1	2	3	2	2	2	1	5	1	3	5	1	3	1	1	3	3	3
25	1	1	2	3	2	2	2	1	1	2	4	5	4	3	1	2	3	4	3
26	1	1	2	3	2	2	2	1	3	1	3	5	4	1	2	2	3	3	4
27	1	1	2	3	1	2	1	1	2	3	3	5	4	5	1	1	4	5	4
28	1	1	2	3	1	2	1	1	1	1	4	5	5	3	1	1	4	5	2
29	1	1	2	3	2	2	2	1	1	1	4	4	5	1	1	1	4	5	2

DP	Site	Sex	Age	Level of education	PS	PE	QSA	WFV	KCF	DOD	DE	ACW	BDC	BuDC	LDCIS	MSCS	TSAOD	TSAED	DTP
30	1	1	2	3	4	3	2	2	2	3	5	4	5	5	1	1	4	5	5
31	1	1	2	3	2	2	1	1	1	1	3	5	5	4	1	2	2	3	3
32	1	1	2	3	1	2	2	1	2	1	4	5	1	4	1	2	2	4	3
33	1	1	2	3	2	2	1	1	1	3	4	4	1	4	1	2	4	5	2
34	1	1	3	3	2	3	1	1	1	2	5	5	3	1	3	1	4	5	5
35	1	1	3	3	2	3	1	1	2	3	4	5	3	1	1	1	4	4	5
36	1	1	3	3	4	3	1	1	1	1	4	5	3	1	1	1	3	5	4
37	1	1	3	3	1	3	1	1	2	1	4	5	3	1	1	1	3	5	5
38	1	1	3	3	1	3	1	1	1	3	5	4	4	1	2	3	3	5	4
39	1	1	3	3	4	3	2	1	3	1	5	4	5	1	2	1	2	5	5
40	1	1	3	3	4	3	2	1	5	3	5	4	5	1	1	1	3	5	4
41	1	1	3	3	2	3	2	1	3	1	5	5	4	1	1	1	2	5	5
42	1	1	3	3	1	2	1	1	1	3	4	5	4	1	1	5	3	5	3
43	1	1	3	4	2	2	2	1	3	5	3	5	3	2	3	5	5	3	3
44	1	1	3	4	1	2	4	1	1	5	4	4	3	2	3	1	5	4	2
45	1	1	3	4	4	2	5	2	3	5	4	5	3	2	1	1	5	5	2
46	1	1	3	4	3	2	5	3	2	5	3	5	2	2	1	1	4	5	4
47	1	1	3	4	4	2	5	2	1	4	3	5	2	1	1	1	4	5	3
48	1	1	3	4	2	2	5	3	2	5	3	5	1	1	3	4	5	5	3
49	1	1	3	4	2	2	5	3	2	3	3	5	1	1	1	1	5	4	3
50	1	1	3	4	2	2	5	3	2	2	3	4	1	1	1	1	5	4	2
51	2	2	1	1	2	1	1	1	2	3	2	5	1	2	3	1	5	2	3
52	2	2	1	1	4	1	1	1	1	5	2	5	5	1	1	4	5	2	3
53	2	2	1	1	2	2	1	1	1	1	3	5	5	1	1	1	5	3	2
54	2	2	1	1	4	1	1	1	1	1	2	5	5	1	1	1	5	2	1
55	2	2	1	1	4	2	1	2	1	3	4	5	5	1	3	1	4	4	1
56	2	2	1	2	2	2	1	1	1	1	4	4	5	1	1	1	3	4	1
57	2	2	1	2	2	2	2	1	1	1	3	5	1	1	1	3	4	3	3
58	2	2	2	2	4	2	4	1	1	1	4	5	3	1	3	3	4	4	4
59	2	2	2	3	4	2	2	1	2	3	4	5	3	1	1	1	3	4	4

DP	Site	Sex	Age	Level of education	PS	PE	QSA	WFV	KCF	DOD	DE	ACW	BDC	BuDC	LDCIS	MSCS	TSAOD	TSAED	DTP
60	2	2	2	3	1	2	1	1	1	1	3	5	4	1	2	1	4	3	3
61	2	2	2	3	1	2	3	1	1	3	3	5	4	3	1	1	3	3	3
62	2	2	2	3	2	2	2	1	1	1	3	4	4	3	1	1	3	3	2
63	2	2	2	3	1	2	1	1	1	1	3	4	4	2	1	1	3	3	3
64	2	2	2	3	1	2	1	1	1	3	3	4	5	2	2	2	4	4	2
65	2	2	2	3	1	2	1	1	2	3	4	4	5	2	1	1	3	4	3
66	2	2	2	3	2	2	1	1	1	1	3	4	5	2	1	1	3	3	2
67	2	2	2	3	2	2	2	1	1	2	4	5	5	1	1	1	3	4	2
68	2	2	2	3	2	2	1	1	5	1	3	5	5	1	2	2	4	3	3
69	2	2	2	3	1	2	1	1	2	1	3	5	4	1	2	1	4	3	3
70	2	1	2	3	2	2	1	1	1	2	3	5	5	1	2	1	4	3	3
71	2	1	2	3	4	2	3	2	1	3	4	4	4	1	1	2	4	4	3
72	2	1	2	3	4	3	4	2	2	1	5	5	5	2	1	2	3	5	5
73	2	1	2	3	4	2	4	1	1	1	4	5	4	2	1	2	3	4	3
74	2	1	2	3	4	2	4	3	1	3	3	5	5	2	1	2	2	4	3
75	2	1	2	3	2	2	3	1	2	1	3	5	4	2	1	1	3	4	2
76	2	1	2	3	2	2	3	1	1	2	3	5	5	1	2	1	3	5	2
77	2	1	2	3	1	2	1	1	1	1	3	5	4	1	1	3	4	3	3
78	2	1	2	3	1	2	1	1	1	3	4	4	3	1	1	1	4	4	2
79	2	1	2	3	1	2	1	1	2	1	4	4	3	2	2	3	4	4	3
80	2	1	2	3	1	2	1	1	1	1	4	4	5	2	1	3	4	4	3
81	2	1	3	3	1	2	1	1	1	1	3	5	5	2	3	1	4	3	4
82	2	1	3	3	1	2	1	1	5	1	4	5	5	1	3	1	4	4	4
83	2	1	3	3	1	2	1	1	1	2	3	5	5	1	1	1	3	3	2
84	2	1	3	3	2	2	1	1	1	1	3	5	2	3	1	4	3	3	3
85	2	1	3	3	2	2	1	1	1	1	3	5	5	3	1	1	3	3	4
86	2	1	3	3	2	2	2	2	1	1	4	4	5	2	2	1	4	4	4
87	2	1	3	3	2	2	2	1	1	1	4	5	1	1	1	1	4	4	4
88	2	1	3	3	2	2	1	1	1	2	4	5	4	1	1	3	4	4	2
89	2	1	3	3	1	3	1	1	1	1	5	5	4	1	1	1	3	5	5

DP	Site	Sex	Age	Level of education	PS	PE	QSA	WFV	KCF	DOD	DE	ACW	BDC	BuDC	LDCIS	MSCS	TSAOD	TSAED	DTP
90	2	1	3	3	2	3	4	3	2	1	5	5	4	1	3	1	3	5	5
91	2	1	3	3	2	3	2	1	1	2	4	5	1	1	1	1	3	5	5
92	2	1	3	3	2	3	2	1	1	1	5	5	1	1	3	4	2	5	4
93	2	1	3	3	2	3	2	1	1	1	5	4	5	1	3	1	2	5	5
94	2	1	3	4	1	3	5	3	2	5	5	5	5	3	3	1	5	5	5
95	2	1	3	4	4	3	4	3	1	5	5	5	5	3	1	3	5	5	4
96	2	1	3	4	1	2	3	2	3	5	4	5	5	2	1	1	5	4	5
97	2	1	3	4	2	2	2	3	3	5	4	5	4	2	2	1	5	4	2
98	2	1	3	4	4	2	5	2	1	5	3	5	4	1	1	1	4	3	2
99	2	1	3	4	1	2	3	3	1	4	3	5	4	1	2	5	5	3	4
100	2	1	3	4	1	2	4	2	3	3	4	5	3	1	2	1	5	4	4
101	3	2	1	1	2	1	1	1	3	1	2	5	3	3	2	1	1	2	1
102	3	2	1	1	4	1	4	3	1	2	2	5	5	3	2	1	1	2	1
103	3	2	1	1	3	1	5	1	1	1	2	5	5	3	1	1	2	2	1
104	3	2	1	1	2	1	2	1	1	1	2	5	4	3	1	1	1	2	1
105	3	2	2	1	2	1	2	1	5	3	3	3	3	1	1	4	2	3	2
106	3	2	2	1	2	1	2	1	1	1	3	3	4	1	1	4	2	3	1
107	3	2	2	2	4	1	2	1	3	3	3	5	4	1	1	1	3	3	1
108	3	2	2	2	4	1	3	1	1	2	2	3	2	1	1	1	3	2	2
109	3	2	2	2	3	1	5	3	1	1	3	5	4	1	1	1	3	3	1
110	3	2	2	2	4	1	5	1	1	1	2	5	4	1	1	1	3	2	1
111	3	2	2	2	4	1	1	1	1	1	3	3	1	2	1	1	4	3	1
112	3	2	2	3	4	1	2	1	1	2	3	5	5	2	1	1	4	3	1
113	3	2	2	3	3	2	2	1	2	3	4	5	5	5	1	1	4	4	3
114	3	2	2	3	2	2	3	1	1	1	4	5	5	1	2	3	3	4	3
115	3	2	2	3	2	2	2	1	1	1	4	2	5	1	1	3	3	4	2
116	3	2	2	3	2	2	2	1	1	2	3	2	5	1	1	1	3	3	2
117	3	2	2	3	2	2	2	1	1	1	3	2	4	1	1	1	4	3	3
118	3	2	2	3	4	2	4	1	2	1	4	3	5	4	2	1	4	4	4
119	3	2	2	3	4	2	1	1	1	3	3	5	5	4	1	1	4	3	4

DP	Site	Sex	Age	Level of education	PS	PE	QSA	WFV	KCF	DOD	DE	ACW	BDC	BuDC	LDCIS	MSCS	TSAOD	TSAED	DTP
120	3	2	2	3	4	2	1	1	1	1	4	5	2	4	1	1	4	4	4
121	3	2	2	3	2	2	1	1	2	1	4	4	4	1	1	2	3	4	3
122	3	2	2	3	2	2	3	1	1	1	3	5	5	1	2	1	3	3	2
123	3	2	2	3	4	2	3	1	1	1	3	5	5	1	2	1	4	3	3
124	3	2	2	3	4	2	2	1	1	1	3	4	5	3	2	1	3	3	2
125	3	1	2	3	2	1	2	1	2	1	2	5	1	3	1	2	4	2	1
126	3	1	2	3	2	1	2	1	1	1	2	5	4	2	1	2	3	2	1
127	3	1	2	3	3	1	5	3	1	2	3	2	5	2	1	2	3	3	1
128	3	1	2	3	4	2	2	1	1	1	3	5	5	1	1	1	4	3	1
129	3	1	2	3	2	2	2	1	2	1	4	5	5	1	3	1	4	4	1
130	3	1	3	3	4	1	4	2	5	1	2	5	4	1	1	1	4	2	2
131	3	1	3	3	1	2	1	1	1	1	4	4	5	1	1	3	4	4	3
132	3	1	3	3	2	1	1	1	1	3	2	4	5	1	2	3	4	2	1
133	3	1	3	3	2	2	1	1	2	1	4	4	3	1	1	3	3	4	4
134	3	1	3	3	2	1	1	1	1	1	2	5	5	1	1	3	3	2	1
135	3	1	3	3	2	2	1	1	1	3	4	5	5	1	1	3	3	4	4
136	3	1	3	3	1	2	1	1	2	1	4	5	5	1	3	3	3	4	4
137	3	1	3	3	1	2	1	1	1	2	4	3	5	5	1	1	3	4	3
138	3	1	3	3	4	2	5	2	1	1	4	3	5	5	1	1	2	4	3
139	3	1	3	3	2	2	4	1	1	3	3	5	3	2	2	1	3	3	2
140	3	1	3	3	2	2	3	1	1	1	3	5	5	2	1	1	3	3	2
141	3	1	3	3	4	2	3	1	1	1	3	4	5	2	1	2	3	3	3
142	3	1	3	3	1	3	1	1	1	1	5	5	4	1	3	1	2	5	5
143	3	1	3	3	4	3	2	2	1	2	5	2	5	1	3	1	2	5	5
144	3	1	3	3	2	3	2	1	1	1	4	5	5	1	1	4	3	4	5
145	3	1	3	3	3	2	5	3	1	1	3	5	5	1	1	1	3	3	4
146	3	1	3	3	1	2	1	1	1	1	3	2	1	1	2	1	3	3	3
147	3	1	3	4	1	2	2	2	3	5	4	5	5	1	3	2	5	4	4
148	3	1	3	4	2	2	5	3	2	5	4	2	5	2	1	1	5	4	4
149	3	1	3	4	4	2	5	2	3	5	3	5	5	1	1	1	5	3	2

DP	Site	Sex	Age	Level of education	PS	PE	QSA	WFV	KCF	DOD	DE	ACW	BDC	BuDC	LDCIS	MSCS	TSAOD	TSAED	DTP
150	3	1	3	4	4	2	5	3	1	5	3	5	5	1	3	3	5	3	2
151	4	2	1	1	1	1	1	1	1	1	2	3	5	1	2	1	2	2	1
152	4	2	1	1	1	1	1	1	5	3	2	5	5	1	1	1	1	2	1
153	4	2	2	1	1	1	1	1	1	1	3	5	5	1	1	2	2	3	2
154	4	2	2	2	1	2	1	1	1	1	4	4	1	1	1	3	3	4	3
155	4	2	2	2	1	2	1	1	1	1	4	5	4	3	1	1	3	4	4
156	4	2	2	2	1	2	1	1	1	1	3	5	4	3	1	1	3	5	4
157	4	2	2	2	1	2	1	1	1	1	3	5	4	1	1	2	3	3	3
158	4	2	2	2	1	2	1	1	1	2	3	5	4	1	2	3	4	5	2
159	4	2	2	3	2	2	1	1	1	1	3	5	2	1	1	1	4	3	2
160	4	2	2	3	2	2	1	1	1	1	3	4	3	4	1	1	4	5	2
161	4	2	2	3	2	2	2	1	1	1	4	5	2	1	1	1	4	5	3
162	4	2	2	3	4	2	2	1	2	2	3	2	2	2	1	4	4	5	3
163	4	2	2	3	4	2	1	1	2	2	4	5	2	2	3	1	4	4	3
164	4	2	2	3	4	2	1	1	3	1	3	5	4	3	3	1	4	5	3
165	4	2	2	3	2	2	1	2	1	1	4	3	5	1	1	4	4	4	4
166	4	2	2	3	2	2	1	1	1	1	3	3	5	1	1	1	4	5	4
167	4	2	2	3	2	2	1	1	1	3	4	5	5	1	2	1	3	4	3
168	4	2	2	3	2	2	3	3	1	1	3	5	5	1	2	1	3	3	4
169	4	1	2	3	2	2	5	3	2	1	3	5	5	2	1	1	3	3	4
170	4	1	2	3	2	2	4	1	5	2	3	5	4	2	1	4	3	5	3
171	4	1	2	3	1	2	2	1	2	1	3	4	5	2	1	4	4	5	4
172	4	1	2	3	2	2	2	1	2	3	4	5	3	2	1	1	3	5	3
173	4	1	2	3	2	2	1	1	1	1	4	5	5	1	2	1	4	4	2
174	4	1	2	3	1	2	1	1	1	1	4	5	3	1	1	1	3	5	3
175	4	1	2	3	1	2	1	1	1	1	4	3	2	1	1	3	3	5	3
176	4	1	2	3	1	2	1	1	1	1	4	5	3	1	1	1	4	4	3
177	4	1	2	3	1	2	1	1	2	1	3	5	3	3	2	1	4	5	2
178	4	1	2	3	2	2	1	1	1	1	3	4	5	1	2	1	4	3	4
179	4	1	2	3	2	2	1	1	1	1	3	5	4	1	2	3	3	5	4

DP	Site	Sex	Age	Level of education	PS	PE	QSA	WFV	KCF	DOD	DE	ACW	BDC	BuDC	LDCIS	MSCS	TSAOD	TSAED	DTP
180	4	1	2	3	2	2	1	1	3	2	3	5	4	1	1	1	3	5	2
181	4	1	2	3	2	1	1	1	1	3	2	4	5	1	1	1	3	2	1
182	4	1	2	3	2	1	1	1	1	1	2	5	1	3	1	1	4	2	1
183	4	1	3	3	2	1	3	1	1	1	2	5	4	1	2	3	3	2	1
184	4	1	3	3	2	1	3	3	1	1	3	3	5	1	1	3	4	3	1
185	4	1	3	3	1	1	1	1	1	1	2	5	5	1	1	1	3	2	1
186	4	1	3	3	2	1	1	1	3	1	3	2	5	1	1	1	4	3	1
187	4	1	3	3	2	1	1	1	1	1	3	5	2	1	3	1	3	3	1
188	4	1	3	3	2	1	1	1	1	2	2	5	5	1	1	2	4	2	1
189	4	1	3	3	2	1	1	1	1	3	2	3	5	4	1	2	3	2	2
190	4	1	3	3	2	1	2	1	3	1	2	5	5	1	1	1	4	2	1
191	4	1	3	3	2	1	2	1	1	1	2	5	3	1	1	1	3	2	1
192	4	1	3	3	2	1	2	1	1	1	3	5	3	2	1	1	4	3	2
193	4	1	3	3	2	1	4	2	1	1	3	3	5	2	2	1	4	3	1
194	4	1	3	3	2	2	2	1	1	1	3	5	5	1	1	2	3	5	1
195	4	1	3	4	2	1	5	2	4	5	3	3	5	1	1	2	1	3	1
196	4	1	3	4	2	1	5	3	5	5	2	5	5	1	1	1	1	2	1
197	4	1	3	4	2	1	4	3	5	5	2	2	1	3	1	1	1	2	1
198	4	1	3	4	2	1	3	2	3	5	2	5	1	1	1	1	2	2	2
199	4	1	3	4	1	1	3	3	4	4	2	5	5	1	1	1	1	2	1
200	4	1	3	4	1	1	1	1	1	4	2	5	4	1	1	1	2	2	1
